# Cellular fragmentation underlies the immunogenicity of irreversible electroporation‐mediated tumor cell killing

**DOI:** 10.1002/btm2.70102

**Published:** 2025-12-22

**Authors:** Joseph R. Vallin, Brandon J. Burbach, Qi Shao, Fang Zhou, Jacob S. Ankeny, Alessio Giubellino, Yoji Shimizu, Samira M. Azarin

**Affiliations:** ^1^ Department of Chemical Engineering and Materials Science University of Minnesota Minneapolis Minnesota USA; ^2^ Department of Laboratory Medicine and Pathology University of Minnesota Minneapolis Minnesota USA; ^3^ Center for Immunology University of Minnesota Minneapolis Minnesota USA; ^4^ Department of Neurosurgery University of Minnesota Minneapolis Minnesota USA; ^5^ Characterization Facility College of Science and Engineering, University of Minnesota Minneapolis Minnesota USA; ^6^ Department of Surgery, Division of Surgical Oncology University of Minnesota Minneapolis Minnesota USA; ^7^ Masonic Cancer Center University of Minnesota Minneapolis Minnesota USA

**Keywords:** dendritic cells, in situ vaccination, irreversible electroporation, melanoma, microparticles, pancreatic cancer, phagocytosis

## Abstract

Irreversible electroporation (IRE) is a focal ablative cancer therapy that destroys cells through membrane destabilization via pulsed electric fields. It also has the capacity to induce a systemic, anti‐tumor immune response, thus acting as an in situ vaccine. Although many studies characterize the immunogenicity of focal therapies by their released biochemical constituents, here we show that the biophysical context of the presentation of these immunogenic signals is vital to understanding downstream immune functions. Compared to thermal ablation or cryoablation, IRE generates similar numbers of exosome‐like particles (ELP, 50–200 nm) but significantly greater numbers of microparticles (MP, 200–1000 nm) and large debris particles (LDP, 2–6 μm) in both melanoma and pancreatic cancer cell lines. We show that LDPs contain antigen and tumor‐associated DNA, which dendritic cells (DCs) internalize in greater proportions from IRE‐treated cells compared to other treatments. For the submicron particles, we demonstrate both in vitro and in vivo that MPs induce greater T‐cell proliferation and differentiation compared to ELPs on a per‐particle basis. This novel biophysical analysis of the immunogenicity of IRE‐treated cancer cells opens a new avenue toward improving the systemic immune response to focal ablation‐based cancer immunotherapies via increasing cell fragmentation and particle generation.

## Graphical abstract

Irreversible electroporation (IRE) of cancer cells generates high amounts of large particulate antigen (>200 nm) in contrast to temperature‐based ablation methods that generate primarily small particulate antigen (<200 nm). The biophysical context of this antigen potentiates the immunogenicity of IRE, leading to increased dendritic cell antigen uptake and T‐cell proliferation.

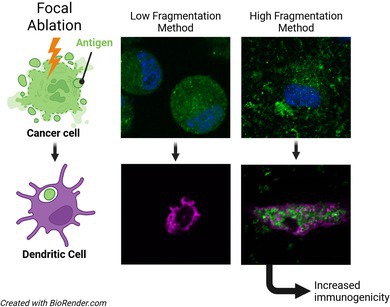

**Translational Impact Statement**

This work augments the current understanding of the mechanism of efficacy for ablation‐based cancer therapies and should provide clinicians with actionable knowledge to improve immune responses via a choice of treatment parameters that maximizes cellular fragmentation.

## 1 INTRODUCTION

Focal ablation cancer therapies encompass energy‐mediated methods for minimally invasive and spatially precise destruction of tumors. Beyond tumor debulking, these therapies have the potential to stimulate a systemic anti‐tumor adaptive immune response through the release of endogenous antigen and danger signals into the tumor microenvironment (TME). Thus, they can broadly be classified as in situ autologous cancer vaccines and, as such, offer an elegant and efficient manner of inducing tumor‐specific immunity since they preclude the necessity for tumor antigen identification and ex vivo recombinant expression and isolation.^1^, ^2^, ^3^ Additionally, unbiased release of tumor proteins allows for “antigen spreading” and a polyclonal T‐cell attack rather than a monoclonal response induced by ex vivo administration of predetermined antigen, which can lead to antigen escape. Amidst a constellation of focal therapies that rely on heating or freezing the tumor, irreversible electroporation (IRE) is unique in that it induces cell death non‐thermally by creating irreversible cell membrane pores using pulsed electric fields. IRE has gathered attention in recent years for its ability to trigger a tumor inhibitive immune response,^4^, ^5^ synergize with adjuvant or checkpoint blockade therapy,^6^, ^7^, ^8^, ^9^, ^10^ and spare sensitive tissue architecture.^11^, ^12^

The diverse immune adjuvant properties of IRE are proposed to be rooted in its induction of immunogenic cell death. Depending on the strength of the electric field, cell death proceeds in an unprogrammed necrotic manner as well as by more delayed processes like necroptosis and pyroptosis, which are held to be highly immunogenic.^13^, ^14^, ^15^, ^16^, ^17^ Studies have observed the release of numerous damage associated molecular patterns (DAMPs) including ATP, high mobility group box protein 1 (HMGB1), and calreticulin.^18^, ^19^ These molecules recruit dendritic cells (DCs), activate them, and mark tumor debris to be phagocytosed by them, respectively. DAMP release leads to the recruitment of F4/80^+^ macrophages 12–24 h post‐ablation and migratory CD103^+^ DCs after approximately 3 days.^20^, ^21^, ^22^ An influx of previously primed T cells appears within 24 h, with another wave entering after 7 days, representing those newly primed with the bolus release of antigen.^21^ Cytotoxic T cells are necessary for the efficacy of IRE, as indicated by several CD8^+^ T‐cell depletion studies.^18^, ^22^, ^23^ Additionally, IRE transiently relieves immunosuppression by decreasing the numbers of regulatory T cells (Tregs) and exhausted PD1^+^ T cells in both the tumor and spleen.^18^, ^24^

Despite this knowledge of the immune response to IRE, little is known about the initial encounter between DCs and cellular debris. The way in which differing ablation methods uniquely affect DC uptake and processing of antigenic debris represents a significant knowledge gap in the field. We hypothesize that the optimal application of focal therapy not only releases sufficient DAMPs and antigen but also packages them in a physiologically relevant biophysical context to optimize DC uptake. For example, it is known that particulate antigen (0.5–1.5 μm) is taken up by DCs up to 10^4^‐fold more efficiently than soluble antigen, but also that 200–300 nm particles are internalized in DCs much faster than 2–4 μm particles.^25^, ^26^ In this study, we analyzed the morphological characteristics of IRE‐treated cancer cells and assessed their impact on DC uptake and downstream T‐cell activation. In contrast to temperature‐controlled modalities like thermal ablation and cryoablation, IRE generates a debris pattern marked by extensive cellular fragmentation, resulting in the generation of large debris particles (LDP, 2–6 μm) and microparticles (MP, 200–1000 nm) in several melanoma and pancreatic cancer cell lines. These features lead to enhanced DC uptake of tumor antigen and T‐cell differentiation. Our results suggest that the biophysical landscape of cancer cell debris significantly affects the quality of the resulting innate and adaptive immune responses. To our knowledge, this study is the first of its kind to analyze the importance of particulate antigen, which has long been appreciated in traditional vaccinology, in the context of tumor ablation methods. While soluble signals are commonly assessed markers of immunogenicity, a more comprehensive analysis of debris particle size and biophysical presentation of signals is an overlooked and critical component of designing ablation‐based in situ cancer vaccines.

## 2 MATERIALS AND METHODS

### 2.1 Cell culture

B16‐F10 cells were obtained from American Type Culture Collection. B16‐ZsGreen (B16‐ZsG) and B16‐OVA‐ZsGreen (B16‐OZ) cells were kindly provided by Max Krummel (University of California San Francisco). KPC‐456, MiaPaCa‐2, and Panc‐1 cells were kindly provided by Paolo Provenzano (University of Minnesota). KPC‐ZsGreen (KPC‐ZsG) and KPC‐OVA‐GFP (KPC‐OG) cells were kindly provided by David DeNardo (Washington University in St. Louis). AsPC‐1 cells were kindly provided by John Bischof (University of Minnesota). AsPC‐1 cells were cultured in RPMI 1640 with L‐glutamine (MilliporeSigma) with 10% FBS (Gibco) and 1% penicillin–streptomycin (Gibco), while all other cell lines were cultured in DMEM high glucose (MilliporeSigma) with 10% FBS and 1% penicillin–streptomycin. Bone marrow‐derived DCs (BMDCs), primary mouse splenic DCs, and primary mouse T cells were cultured in suspension using T‐cell proliferation media (TCPM): RPMI 1640 w/o L‐glutamine (Corning), 10% fetal bovine serum (Gibco), 1% penicillin–streptomycin (Gibco), 1% GlutaMAX (100×) (Gibco), 1% HEPES (Gibco), 1% 100 mM sodium pyruvate (Gibco), 1% MEM nonessential amino acids (100×) (Gibco), and 0.05% 50 mM 2‐mercaptoethanol (Gibco).

### 2.2 Focal ablation of cancer cells

Cancer cells were treated according to previously established protocols, which were optimized to ensure complete cell death.^27^ Cancer cells at approximately 90% confluency were lifted with 0.25% trypsin–EDTA (Gibco), centrifuged for 5 min at 300 g, and resuspended in PBS. For thermal ablation (Heat), the cell suspension was placed in a 50°C water bath for 30 min. For cryoablation (Cryo), cells were placed in a pre‐cooled methanol bath for 30 min at −80°C, followed by a passive thaw to room temperature. For IRE, the cell suspension was loaded into a 4 mm electroporation cuvette (BTX) and electroporated using an ECM 830 electrical pulse generator (BTX/Harvard Apparatus) at 1250 V/cm for 99 square wave pulses of 50 μs at a 1 Hz frequency. Focally ablated cells were either used as is or centrifuged for 10 min at 200 g to recover the supernatant, which we refer to as the ablation supernatant. For IRE treatment of adherent cells, see Data S1, Supporting Information.

### 2.3 Quantification of cellular fragmentation

Ablated cells were imaged with brightfield microscopy using an EVOS FL Auto microscope (ThermoFisher Scientific). ImageJ (version 1.54e) was used for image thresholding and to count and determine the size of each particle in the ablated cell preparation. The area‐weighted size distribution was determined by calculating the percentage of the total area of cellular debris for particles in each bin. To characterize submicron debris generation, ablation supernatants were diluted in PBS and analyzed with nanoparticle tracking analysis (NTA) using a NanoSight LM10 (Malvern Panalytical, UK) equipped with a 400 nm laser. To analyze cellular debris with transmission electron microscopy (TEM), ablation supernatants or control extracellular vesicles (EVs, derived via differential centrifugation of conditioned media) were placed on glo‐discharged copper/formvar EM grids (Electron Microscopy Sciences) and stained with 1% uranyl acetate (Electron Microscopy Sciences).^28^ They were imaged with a G2 Spirit Bio‐TEIN TEM (Technai) at 120 kV of accelerating voltage.

### 2.4 Immunocytochemical analysis of focally ablated cancer cells

For immunostaining of plasma membrane and DNA localization, ablated cells (1 × 10^6^ cells/mL) or control non‐ablated cells were stained with 5 μg/mL wheat germ agglutinin Oregon Green 488 conjugate (Invitrogen) or 4 μM EthD‐1 (Invitrogen) in PBS for 10 min at 37°C or 30 min at room temperature, respectively, and analyzed with an EVOS FL Auto microscope. For immunostaining of antigen and DAMPs, ablated cells (1 × 10^6^ cells/mL) and non‐ablated control cells were centrifuged onto microscope slides with a Shandon Cytospin 4 cytocentrifuge (ThermoFisher Scientific) for 3 min at 1000 RPM. Samples were immediately fixed and stained using standard methods described in Data S1 and then imaged using an EVOS FL Auto microscope or an A1Rsi HD confocal microscope (Nikon).

Cellular release of dsDNA was quantified with the Quant‐iT PicoGreen dsDNA Assay kit (Invitrogen) according to the manufacturer's instructions. Ablation supernatants, or supernatants from untreated cells or RIPA buffer (ThermoFisher Scientific) treated cells (negative and positive controls, respectively), were incubated in 100 μg/mL RNase A (Cell Signaling) and 0.75 U/μL S1 Nuclease (Promega) for 30 min at 37°C to degrade RNA and ssDNA, respectively, prior to analysis with the assay kit.

### 2.5 Histological analysis of in vivo IRE‐treated KPC tumors

All animal studies adhered to ARRIVE guidelines and were performed in accordance with the Institutional Animal Care and Use Committee (IACUC) of the University of Minnesota (protocol 2312‐41574A). For in vivo experiments, female mice were used to enable mixing and randomization of groups while preventing fight wounds, which would confound results. Previous experience with similar experiments in male mice suggests that results from these studies should be generalizable to both genders. Female wild‐type C57BL/6J mice obtained from The Jackson Laboratory were injected subcutaneously on the right flank with 2.5 × 10^5^ KPC‐456 cells in 50 μL of Matrigel (Corning). After 17 days, round tumors had grown to about 3–4 mm in diameter. Mice were anesthetized with ketamine (100 mg/kg) and xylazine (10 mg/kg) via intraperitoneal injection, and each tumor was ablated with IRE using 5 mm platinum tweezer‐style electrodes (Tweezertrode, BTX) connected to a square wave pulse generator (ECM 830, BTX) that administered a dose of 1250 V/cm for 99 pulses of 50 μs at a 1 Hz frequency. One mouse was left untreated as a sham control. At various timepoints, the tumors were excised and processed into formalin‐fixed and paraffin‐embedded tissue sections that were stained with hematoxylin and eosin (H&E). Slides were imaged using an Olympus BX40 microscope equipped with Olympus cellSens software (version 4.3) and were scored for viability by a Board‐certified anatomic pathologist. Tumor cells in the sections were classified as viable (preserved nuclear and cytoplasmic features, as seen in the control tissue), intermediate (cells with preserved nucleus, but with signs of cell stress, including loss of cohesion due to detachment from adjacent cells, shrinkage, and cell condensation), and necrotic cells (characterized by irreversible condensation of the nucleus/pyknosis, karyolysis/karyorrhexis, or complete loss of nucleus, and degradation of cytoplasmic features).

### 2.6 Dendritic cell isolation

BMDCs were obtained from the bone marrow of the femur and tibia of C57BL/6 mice. Singularized bone marrow cells were treated with ACK lysis buffer (Gibco) to remove red blood cells and seeded in non‐treated six‐well plates to a concentration of 2 × 10^6^ cells/mL in TCPM supplemented with 100 ng/mL of Flt3‐ligand (Flt3‐L). Non‐adherent and loosely adherent BMDCs were harvested after 9 days.

Alternatively, DCs were acquired from the spleens of mice injected with Flt3‐L expressing B16‐F10 cells^29^ (provided by Marc Jenkins, University of Minnesota). Briefly, C57BL/6 mice were injected with 5 × 10^5^ B16‐Flt3‐L cells in the right flank and were sacrificed 2–3 weeks later once a 5–10 mm tumor had formed. The harvested spleens were processed into a single cell suspension as described elsewhere,^8^ and DCs were isolated via magnetic bead‐based negative selection. Herein, we refer to these DCs as splenic DCs.

### 2.7 Assessing DC uptake of cellular debris

DC uptake of tumor‐derived DNA was assessed using the Click‐iT EdU Cell Proliferation Kit (ThermoFisher Scientific) according to the manufacturer's instructions. Briefly, ablation supernatants of EdU‐labeled B16‐F10 cells were mixed with 1.2 × 10^5^ BMDCs in a 96‐well plate. After 2 h, samples were treated with 50 U/mL DNase I (Qiagen) for 15 min to degrade residual extracellular DNA. The samples were deposited onto microscope slides as above, fixed, and stained with EdU‐labeling azide‐conjugated AF647 and Super Bright 436 conjugated anti‐MHCII (Invitrogen). Slides were mounted and imaged on a Nikon A1Rsi confocal microscope. To quantify DNA uptake, ablation supernatants of EdU‐labeled B16 cells were seeded into a 96 well plate along with 1.5 × 10^5^ splenic DCs in TCPM. After 2 or 4 h of incubation, the cells were lifted, stained with flow antibodies (see Table S1) and azide‐AF647, and analyzed by flow cytometry.

To assess DC uptake of antigenic debris, ablated KPC‐ZsG or B16‐OZ cells (1.5 × 10^5^ cell equivalents) expressing the fluorescent construct ZsGreen were seeded into a Lab‐Tek II 8‐chamber glass slide (ThermoFisher Scientific) with 1.5 × 10^5^ BMDCs in TCPM. Cell equivalents indicate cell count prior to ablation. After 3 or 8 h, the slide was centrifuged for 5 min at 300 g, the media was aspirated, and the cells were fixed with 4% PFA for 15 min at room temperature, blocked with 5% rat serum (STEMCELL Technologies), and stained with 1:50 diluted Super Bright 436 conjugated anti‐MHCII overnight at 4°C. Samples were imaged with a Nikon A1Rsi confocal microscope. To quantify uptake, 1.5 × 10^5^ cell equivalents of ablated KPC‐ZsG cells were added to 1.5 × 10^5^ BMDCs in a 96 well plate. Alternatively, B16‐F10 cells were stained with 30 μM PKH26 using the PKH26 Red Fluorescent Cell Linker Kit (MilliporeSigma) following manufacturer instructions, treated with focal ablation, and added to BMDCs at the same titer. For negative controls, an equivalent volume of PBS or 1.5 × 10^5^ unstained ablated B16‐F10 cells were added per well. For a positive activation control, polyinosinic‐polycytidylic acid (poly(I:C)) (InvivoGen) was added for a final concentration of 5 μg/mL. BMDCs were incubated with ablated cancer cells for 3–24 h, stained, and then analyzed with flow cytometry.

To measure phagocytosis inhibition, BMDCs were treated with 0–10 μM of cytochalasin D (MilliporeSigma) for 1 h at a cell density of 6 × 10^5^ cells/mL. The BMDCs were transferred to a 96‐well plate (250 μL per well) and pulsed with 50 μL of ablated PKH26‐stained B16‐F10 cells (3 × 10^5^ cell equivalents/mL) for three additional hours before analysis by flow cytometry.

### 2.8 Microparticle and exosome‐like particle isolation

Exosome‐like particles (ELPs) were isolated from IRE ablation supernatants via differential centrifugation. IRE ablation supernatant was centrifuged sequentially at 2000 g for 10 min, 10,000 g for 90 min, and 100,000 g for 70 min. The ELPs pelleted in the last spin were resuspended in approximately 150 μL of PBS. MPs were isolated from IRE ablation supernatants via syringe filtration. A 1 mL syringe was used to pass IRE ablation supernatant through a pre‐wetted 0.22 μm syringe filter (MilliporeSigma) at a rate of approximately 10 μL/s. Then, 2 mL of PBS was flushed through the filter in the same direction and flow rate. Finally, 2–3 mL of PBS was rapidly flushed through the filter in the opposite direction. The collected retentate was centrifuged at 10,000 g for 90 min and resuspended in approximately 150 μL of PBS. Aliquots of ELP and MP suspensions were analyzed with NTA for particle size and concentration.

### 2.9 T‐cell activation and proliferation

T cells were harvested from the lymph nodes and spleen of male or female transgenic OT‐I T‐cell receptor mice following a negative immunomagnetic selection protocol^30^ and then stained with cell trace violet^27^ (CTV, Invitrogen) at 37°C in 5% FBS in PBS with 4.2 μM CTV for 15 min for in vitro use or 8.4 μM CTV for 7.5 min for in vivo use. For in vitro studies, BMDCs were incubated for 24 h in a 96‐well plate at 1.5 × 10^5^ cells/well in TCPM containing 5 μg/mL of poly(I:C) along with equal particle numbers of either ELPs or MPs. Then 5 × 10^4^ CTV^+^ OT‐I T cells were added per well for 72 h. Cells were analyzed with flow cytometry. For in vivo studies, 5 × 10^5^ CTV+ OT‐I T cells were transferred into female C57BL/6 mice via retro‐orbital injection, and several hours later, mice were injected subcutaneously with ELPs and/or MPs into the right flank. As negative and positive controls, mice were injected with sham PBS or 5 × 10^5^ live B16‐OZ cells, respectively. After 2–3 days, the mice were euthanized, and the spleen and both inguinal lymph nodes were harvested, processed into a single‐cell suspension following previously described methods,^8^ and analyzed with flow cytometry.

### 2.10 Flow cytometry

Cells were washed with FACS buffer (2% FBS in PBS) and stained with flow antibodies (see Table S1) for 30 min. Cells were washed again with FACS buffer, resuspended in 0.5% PFA, and a known number of PKH counting beads (MilliporeSigma) were added to each sample to permit quantification of total cell numbers. Flow cytometry was performed using a BD LSRII, BD Fortessa X‐20, or a BD Symphony A3 cytometer. Data were analyzed using FlowJo software version 10.8.1. The T‐cell proliferation index was calculated using the Proliferation Modeling tool on CTV histogram plots of gated OT‐I T cells.

### 2.11 Statistical analysis

Statistical analysis was performed using GraphPad Prism 9. Specific details are provided in the figure captions. Statistical significance was attributed to *p* values of <0.05.

## 3 RESULTS

### 3.1 Analysis of the morphology and biochemical composition of focally ablated cancer cells

**FIGURE 1:**
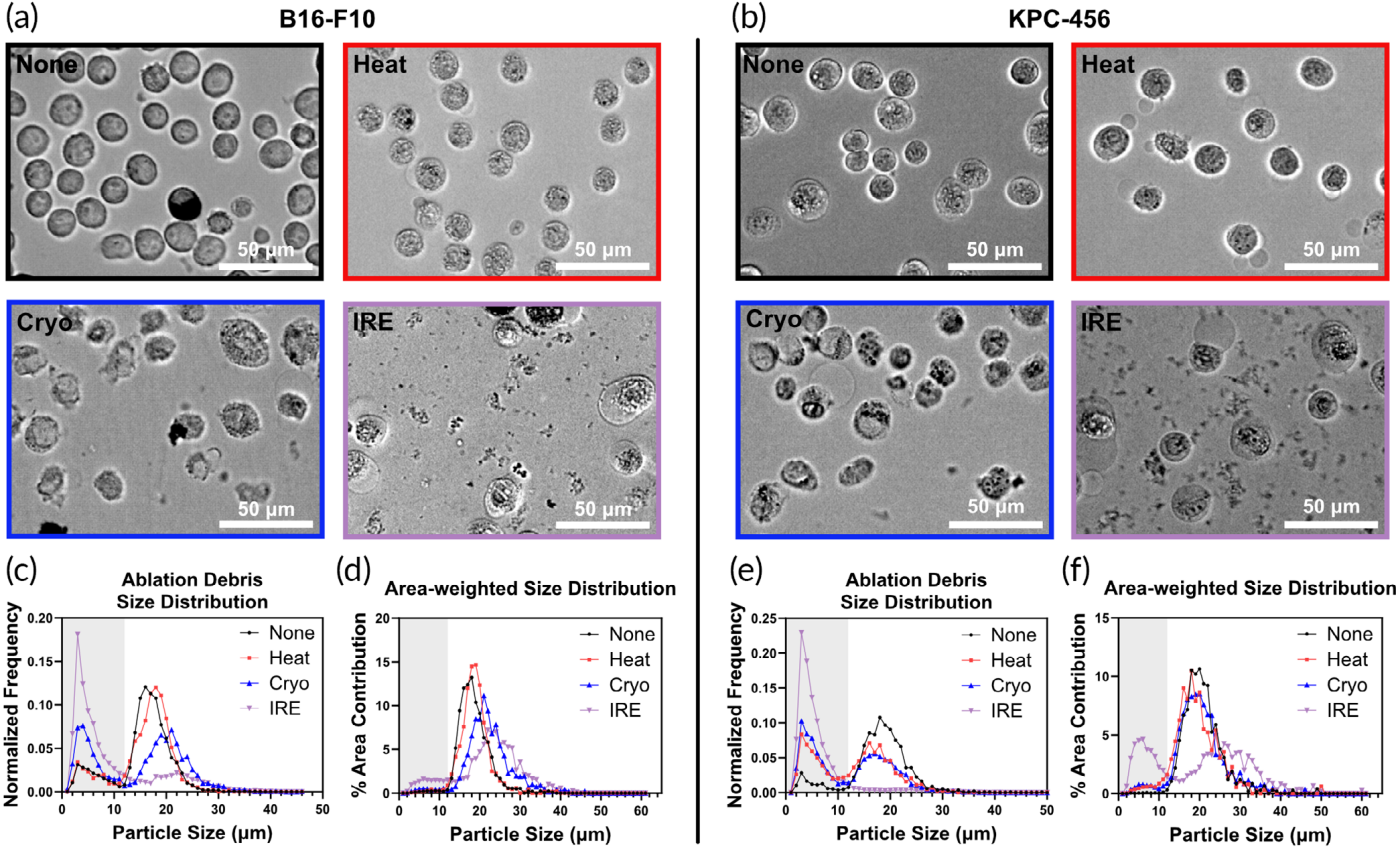
IRE‐treated cancer cells display a fragmented morphology with increased numbers of LDPs. (a, b) Brightfield micrographs of focally ablated cancer cells. Scale bar: 50 μm. (c, e) Size distribution of particles in the cellular debris as measured by ImageJ. (d, f) Size distribution of particles weighted by their contribution to the total 2D‐projected area of cellular debris. The region of LDPs is shaded for emphasis. Size distribution data aggregated from four representative images of each sample (see Figure S1).

B16‐F10 melanoma cells and KPC‐456 pancreatic cancer cells were treated in suspension with several common methods of focal ablation: thermal ablation (Heat), cryoablation (Cryo), and IRE. The resulting morphology was examined with brightfield microscopy (Figures 1a,b and S1). Representative images of live untreated cells showed a typical round morphology with very few subcellular particles. Cells treated with Heat displayed a morphology comprised largely of round, non‐disintegrated dead cells with minimal blebbing. Heat‐treated cells were significantly stiffer than Cryo‐treated, IRE‐treated, or non‐ablated cells as they did not fully flatten when pelleted onto a glass slide (Figure S2). Cells treated with Cryo were more irregularly shaped and exhibited a rougher and more serrated surface morphology. Additionally, some cell fragments or vesicular bodies were observed next to the damaged cells. These particles were measured to be 2–6 μm, which we define as LDP hereafter. Finally, IRE‐treated cells displayed a hyper‐fragmented morphology with significantly more LDPs than Cryo. A noticeable portion of the cells that remained intact contained a cell membrane that was detached and distended from the underlying cytoskeleton. This highly fragmented morphology was also observed across three different human pancreatic adenocarcinoma lines (AsPC‐1, MiaPaCa‐2, and Panc‐1) treated with the same dose of IRE (Figure S3). Size distribution analysis of the ablated cell debris fields was used to quantify these visual observations. While intact cells maintained the typical ~18 μm diameter (central peak Figure 1c–f), for both B16‐F10 and KPC‐456 cell lines IRE produced more than double the amount of LDPs compared to Cryo or Heat (leftward peak, shaded for emphasis, Figure 1c,e). For IRE‐treated KPC‐456 cells, LDPs accounted for almost an equal percent of total cellular area as the intact cells (Figure 1f).

**FIGURE 2:**
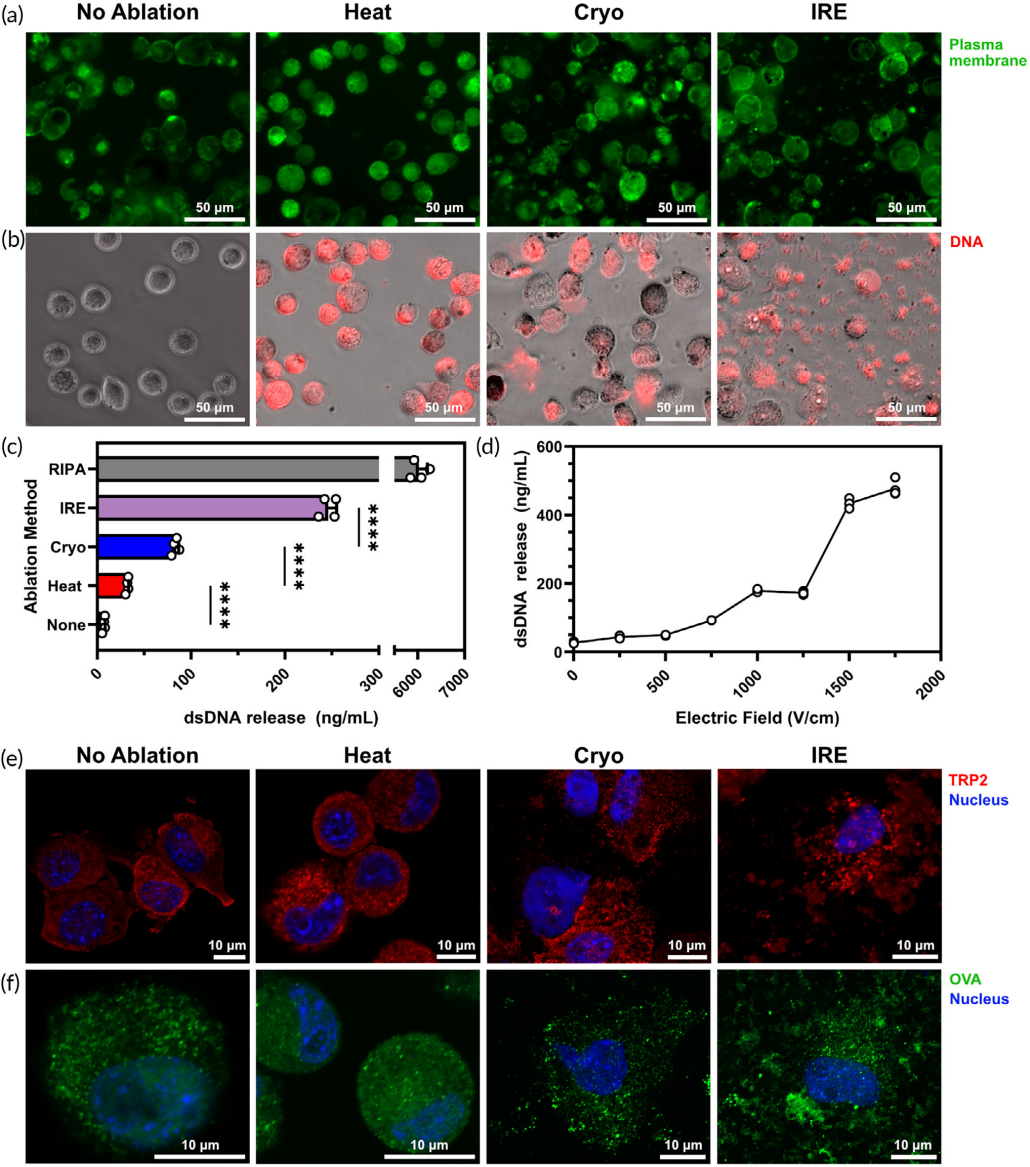
IRE‐derived LDPs have a composition that includes plasma membrane, DAMPs, and antigen. B16‐F10 melanoma cells were ablated with Heat, Cryo, IRE, or left unablated and stained to observe localization of (a) plasma membrane (WGA) and (b) exposed DNA (EthD‐1). EthD‐1 fluorescence images were superimposed on phase micrographs to reveal localization. Scale bar: 50 μm. (c) Quantification of soluble dsDNA released from B16‐F10 cells (1 × 10^6^ cells/mL) by each ablation method compared to a RIPA buffer whole cell lysis control. Error bars display standard deviation (SD). Data analyzed with ANOVA using Tukey's test for multiple comparisons. ****p < 0.0001. (d) Quantification of soluble dsDNA release from B16‐F10 cells (1 × 10^6^ cells/mL) treated with IRE at increasing electric fields. All other parameters held constant at 99 × 50 μs pulses at 1 Hz. Line passes through the mean values. (c, d) *n* = 4 technical replicates, and data shown are from one representative experiment of three independent experiments. (e, f) B16‐F10 cells or B16‐OZ cells were ablated, and the localization of (e) TRP2 tumor antigen or (f) the fluorescent OVA‐ZsGreen model antigen construct was observed, respectively. Scale bar: 10 μm.

Ablated cells were next analyzed for composition and the localization of immunologically relevant biomolecules. A significant portion of LDPs from both Cryo and IRE stained positive for the plasma membrane, revealing the presence of spherical, membranous bodies, and irregularly shaped cell fragments (Figure 2a). We also investigated DNA release from the treated cells, an agonist integral to innate immune detection of tumors through activation of type 1 IFN signaling in DCs via the STING pathway.^31^ For Heat and Cryo samples, the DNA was largely confined to intact cells (Figure 2b). Although Cryo samples did show some signs of DNA expulsion from the cell bodies, this phenomenon was much more apparent in the IRE samples, where nearly all the LDPs contained extracellular DNA. Comparable DNA localization patterns were observed in focally ablated human AsPC‐1, MiaPaCa‐2, and Panc‐1 cells (Figure S4). Similar to DNA, the DAMP HSP70 was only seen in significant amounts on LDPs of IRE‐treated cells (Figure S5). To confirm the increase in extracellular DNA observed for IRE‐treated cells, a fluorescence‐based microplate assay was performed on ablation supernatants to quantify released dsDNA. As hypothesized, IRE supernatants contained threefold more dsDNA than Cryo supernatants and eightfold more than Heat supernatants (Figure 2c). Additionally, the amount of dsDNA released by IRE treatment could be modulated by the strength of the applied electric field (Figure 2d). Extracellular dsDNA increased monotonically with electric field, with a sharp increase starting at the transition from reversible electroporation to IRE after 750 V/cm. This aligns well with other studies that have documented increased DAMP release with increasing electric field.^32^, ^33^

Focally ablated cells were also examined for localization of tumor antigen: the endogenous antigen Tyrosinase‐Related Protein 2 (TRP2) in B16‐F10 cells and the model antigen Ovalbumin (OVA) in transgenic B16‐OVA‐ZsGreen (B16‐OZ) cells (Figure 2e,f). For both cell lines, antigen was entirely localized in the dead cell bodies for Heat samples. In contrast, minor disintegration of the cell periphery was apparent for Cryo samples, with antigen observed on LDPs being released from the compromised cell borders. IRE samples exhibited more extensive cell fragmentation than Cryo, with antigen again present on the disseminated LDPs.

### 3.2 Biophysical features of in vitro IRE‐treated cancer cells are also observed in tumors treated in vivo

**FIGURE 3:**
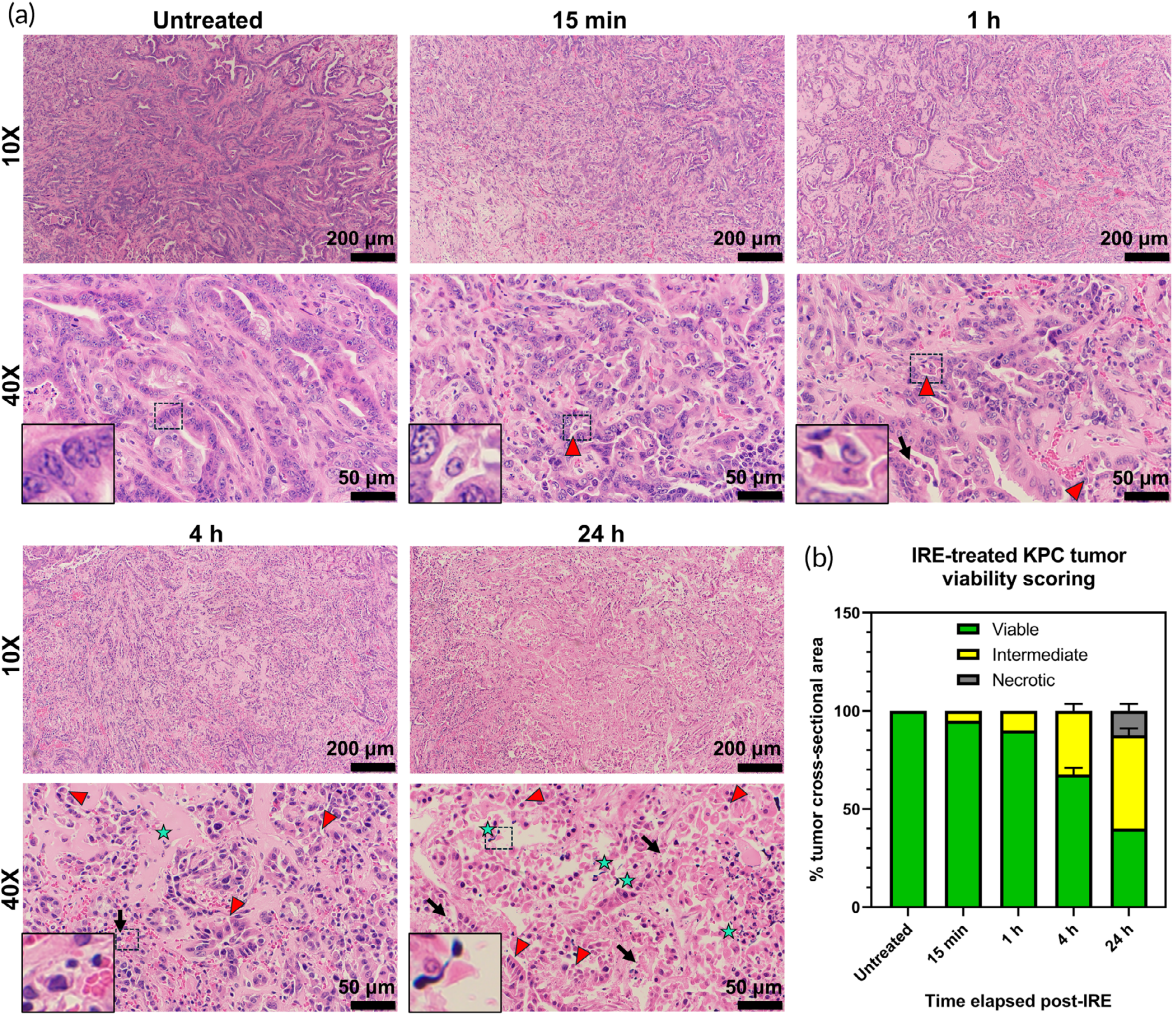
IRE treatment of subcutaneous KPC tumors causes delayed cell death characterized by tissue disaggregation. (a) H&E images of KPC tumors at increasing timepoints following IRE ablation show evidence of discohesive cells (red ▲), generation of LDPs (black →), pyknosis, extracellular DNA (green ★), and eventually full tissue necrosis. Scale bar: 200 μm (10×), 50 μm (40×). Insets on 40× images display a magnified view of the portion outlined in the dotted box. (b) KPC tumors were scored for tissue viability. Two serial tissue sections were scored for each condition. Tissue that was healthy was labeled as viable. Tissue that was showing signs of distress or progressing through cell death was labeled as intermediate. Tissue that showed complete cell death with loss of nuclei was labeled as necrotic.

To determine if IRE‐mediated LDP and DNA release were also observed in a more clinically relevant setting, we first treated B16‐F10 and KPC‐456 cells with IRE in adherent 2D culture, instead of in suspension, and still observed the generation of LDPs (Figure S6). Then, moving to an in vivo pancreatic cancer model, we ablated subcutaneous KPC‐456 tumors in C57BL/6 mice. At varying timepoints following ablation, histological examination of the tumors was performed to understand the early dynamic cellular response to IRE (Figure 3a). Viability scoring of the tumors revealed a continuous increase in the percentage of dying tissue over the first 24 h following IRE treatment (Figure 3b). Healthy, untreated KPC tumors were comprised of an eosinophilic tumor stroma supporting an extensive network of “ducts” lined with basophilic epithelial‐like cells (Figure 3a, Untreated, 40×). After IRE, intermediate cell death, defined by signs of cellular distress (cells with preserved nucleus but with loss of cohesion due to detachment from adjacent cells, shrinkage, and cell condensation), was noted in some areas as early as 15 min post‐ablation, and by 24 h approximately 60% of the total tumor cross‐sectional area was dead (Figure 3b). Cell junction separation (discohesion) was observed in sparse cases by 15 min. By 4 h, this was more prevalent throughout the tumor interior, and by 24 h widespread tissue disaggregation was recognizable along with cases of cellular fragmentation and the release of LDPs into the interstitial space (Figure 3a, 24 h, 40×). Chromatin condensation (pyknosis) was first noted at 1 h and appears common by 4 h. By 24 h, pyknosis appeared to give way to expulsion of chromatin from the cell (Figure 3a, 24 h, 40×) as numerous puncta of extracellular DNA were evident along with large patches of anuclear necrotic tissue (Figure 3a, 24 h, 10×).

### 3.3 Increased cellular fragmentation for IRE treatment is associated with functional differences in dendritic cell performance

**FIGURE 4:**
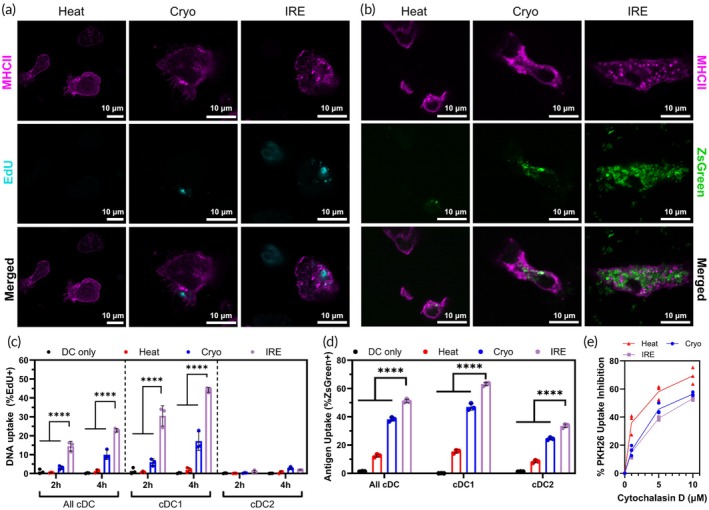
IRE leads to increased dendritic cell uptake of cancer cell‐derived DNA and antigen. (a) Uptake of cancer cell‐derived DNA (EdU) in BMDCs (MHCII). B16‐F10 cells with EdU incorporated into their DNA were treated with focal ablation, and the ablation supernatant was incubated with BMDCs for 2 h and imaged with confocal microscopy. (b) Uptake of tumor‐derived proxy antigen (ZsGreen) in BMDCs. KPC‐ZsG cells were treated with focal ablation and incubated with BMDCs for 8 h and imaged with confocal microscopy. (a, b) Scale bar: 10 μm. (c) Splenic DCs were incubated with ablation supernatants of EdU^+^ B16‐F10 cells for 2 or 4 h and then assayed for uptake of tumor‐derived DNA using flow cytometry. (d) BMDCs were incubated with ablated KPC‐ZsGreen cells for 12 h and then assayed for uptake of ZsGreen using flow cytometry. (c, d) Data are from 1 of 3 independent experiments. Data were analyzed with ANOVA using Tukey's test for multiple comparisons. Error bars display SD. ****p < 0.0001. (e) Percent inhibition of BMDC uptake of ablated, PKH26‐stained B16‐F10 cells caused by increasing concentrations of cytochalasin D. *n* = 3 technical replicates. Lines pass through the mean values. Data representative of two independent experiments.

We hypothesized that the increased fragmentation of IRE‐treated cells would lead to the uptake of more tumor‐derived DNA and antigen, due to their increased partitioning onto particles small enough to be easily phagocytosed by DCs.^34^ To compare DC uptake of released extracellular DNA across the different ablation methods, 5‐ethynyl‐2′‐deoxyuridine (EdU), an analog of thymidine, was incorporated into the DNA of B16‐F10 cells via their culture media (Figure S7). Ablation supernatants of these cells were incubated with bone marrow‐derived DCs (BMDCs) for 2 h. Confocal microscopy revealed that BMDCs did not take up any B16‐F10‐derived EdU^+^ DNA from Heat supernatant (Figures 4a and S8). Cryo supernatant prompted minor DNA uptake, although the EdU puncta appeared to be localized at the cell membrane and not internalized. In contrast, when incubated with IRE supernatant, significantly more BMDCs internalized DNA. For verification, we measured EdU uptake in MHCII^+^, CD11c^+^ splenic conventional DCs (cDCs) after 2 or 4 h of incubation with ablation supernatants via flow cytometry. Splenic DCs are more phagocytic than BMDCs and allowed us to more easily distinguish differences in uptake. At both timepoints, cDCs took up quantifiably more B16‐F10 DNA from IRE supernatant compared to Cryo, and little to no DNA was taken up from Heat supernatant (Figures 4c and S9). Uptake increased with time, and by 4 h, 23% ± 1% of cDCs incubated with IRE supernatants were positive for EdU compared to only 10% ± 3% for Cryo and 1% ± 1% for Heat. Interestingly, for all ablation methods, almost all the EdU^+^ cDCs were from the XCR1^+^, Sirp‐α^−^ cDC1 subtype compared to the XCR1^−^, Sirp‐α^+^ cDC2 subset: for cDCs incubated for 4 h with IRE supernatant, 44% ± 1% of cDC1s were EdU^+^, whereas only 1.9% ± 0.1% of cDC2s were EdU^+^.

Tumor antigen uptake by cDCs was assessed by incubating BMDCs with ZsGreen‐expressing KPC cells (KPC‐ZsG) treated with each ablation method. Observation of the degradation‐resistant fluorescent protein ZsGreen within cDCs was interpreted as a proxy for antigen uptake, which has been demonstrated elsewhere as a reliable system for antigen tracking.^35^ After 8 h of co‐incubation, confocal microscopy revealed that very few BMDCs had taken up puncta of ZsGreen from Heat‐treated cells (Figures 4b and S8), though there were rare instances of BMDCs engulfing entire small dead cells or large (~8 μm) LDPs (Figure S8). Additionally, this ZsGreen did not appear to disseminate throughout the cell's cytoplasm but was constrained in spherical compartments, suggesting that the Heat treatment had induced protein aggregation which impeded antigen processing. Incubation with Cryo‐treated cells led to easily distinguishable uptake of small ZsGreen puncta, which were localized inside the cell membrane. Likewise, BMDCs incubated with IRE‐treated cells also contained ZsGreen in the cytoplasm, but to a notably greater degree. Several of these BMDCs had a cytoplasm that was completely full of diffuse or punctate ZsGreen. Similar results were seen when BMDCs were incubated with ablated B16‐OZ cells (Figure S10). As with the DNA uptake experiment, these results were verified with flow cytometry. After incubating BMDCs with ablated KPC‐ZsG cells for 12 h, the percent of cDCs staining positive for ZsGreen was measured (Figure S11). For IRE, 51% ± 1% of cDCs were ZsGreen^+^ compared to 38% ± 1% for Cryo and 12% ± 1% for Heat (Figure 4d). As with DNA, uptake of ZsGreen was biased toward the cDC1 subtype. For BMDCs incubated with IRE‐treated cells, 63% ± 1% of the cDC1 population was ZsGreen^+^ compared to 34% ± 1% of the cDC2 population.

To validate the ability of the flow cytometry‐based uptake assay to measure the actual uptake of cancer cell DNA and antigen rather than just associations or interactions with the cell membrane that do not lead to successful internalization, we conducted a phagocytosis inhibition assay (Figures 4e and S12). B16‐F10 cells were stained with the cell membrane dye PKH26, ablated, and added to BMDCs in the presence of various concentrations of the phagocytosis inhibitor Cytochalasin D. A dose‐dependent increase in the inhibition of cDC uptake was observed for BMDCs incubated with ablated cells from each method, indicating that a substantial portion of measured uptake was a result of actin‐dependent phagocytosis as opposed to non‐specific binding to the cell membrane. Additionally, the percent of PKH26^+^ cells within various myeloid subtypes matched what would be expected based on their physiological phagocytic capabilities.^36^, ^37^ A significantly higher percentage of F4/80^+^ macrophages took up PKH26^+^ cell debris compared to cDCs, which in turn took up more cell debris compared to Siglec H^+^ plasmacytoid DCs (Figure S12).

### 3.4 Evidence of IRE‐induced cellular fragmentation is also observed on the submicron length scale

**FIGURE 5:**
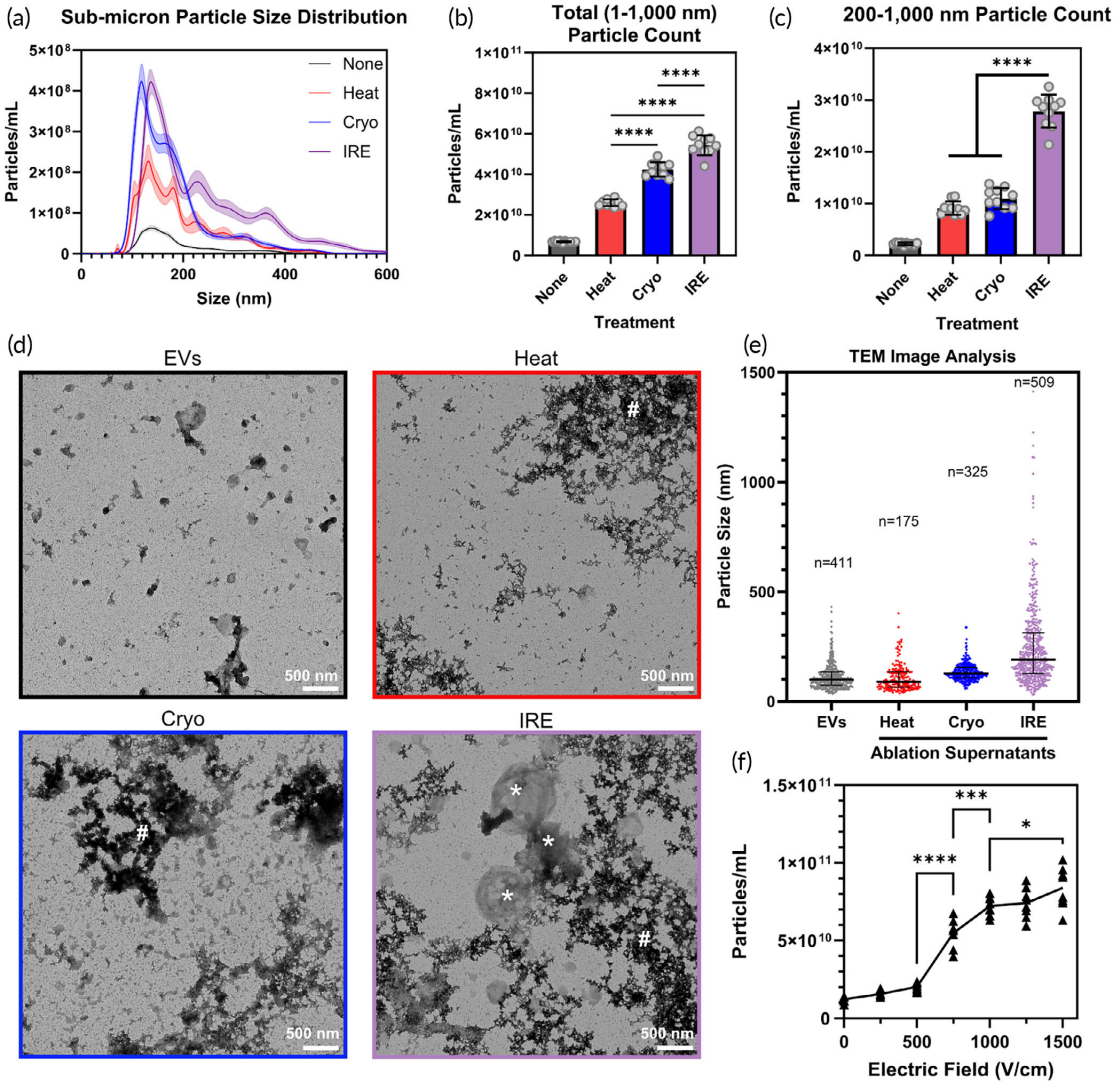
Increased generation of submicron cellular debris is evident in IRE supernatant with the unique presence of large vesicular microparticles. (a) NTA was used to measure the size distribution of submicron cellular debris present in the supernatants of ablated B16‐F10 cells. Line denotes mean and shaded area denotes standard error of the mean (SEM). (b) Total particle concentration in each B16‐F10 ablation supernatant as determined by NTA. Error bars denote SD. (c) Concentration of 200–1000 nm particles in each B16‐F10 ablation supernatant as determined by NTA. Error bars denote SD. (a‐c) Data from one representative experiment of 6 independent experiments, *n* = 10. (d) TEM images of ablation supernatants of B16‐F10 cells (* denotes examples of MPs and # denotes examples of protein aggregates). As a control, naturally derived EVs were isolated from conditioned B16‐F10 cell‐culture media. Scale bar: 500 nm. (e) Particle size distribution as determined by TEM. Error bars indicate median ± inner quartiles. Particle size measurements are aggregated from 3 to 6 images for each ablation method. Each distribution is statistically significant from every other distribution via Tukey's test, *p* < 0.05. (f) Total particle concentration (1–1000 nm), as measured by NTA, of supernatants of B16‐F10 cell suspensions treated with IRE at increasing electric field doses. Data from one representative experiment of four independent experiments. Line passes through the mean values, *n* = 8. (b, c, f) Statistical analysis performed using ANOVA with Tukey's test for multiple comparisons: *p < 0.05, ***p < 0.001, ****p < 0.0001.

Since differences in particle size at a submicron scale have been implicated as a dominant factor affecting DC uptake, we quantified particle number and size in the supernatants of ablated B16‐F10 cells and a non‐ablated control using NTA (Figure 5a). IRE supernatant had the highest total submicron particle concentration compared to Cryo and Heat (Figure 5b). All three ablation methods contain a significant population of particles in the 100–200 nm size range. We termed this subset of particles as ELPs due to their similarity in size, but uncertain similarity in biogenesis, to canonically defined exosomes.^38^ However, when examining 200–1000 nm particles, which fall into the MP range, IRE produced 2.5× and 3.1× more MPs than Cryo and Heat, respectively (Figure 5c). These results were verified by TEM, which revealed that IRE alone contained a significant population of large (200–1000 nm) round membranous structures (several examples marked in the micrographs with an asterisk) (Figure 5d). Excluding the fractal aggregates, likely a mixture of released protein and DNA,^39^ the sizes of all membranous particles in the TEM images were measured, and the resulting size distribution (Figure 5e) matched well with the NTA distribution (Figure 5a). Interestingly, just as extracellular DNA release increased with the electric field of IRE, so did the amount of submicron particles, which also exhibited a sharp jump near the transition from reversible electroporation to IRE around 750 V/cm (Figure 5f). This presents an opportunity for the modulation of downstream anti‐tumor immunological effects through the appropriate choice of IRE operating parameters.

### 3.5 Submicron tumor cell fragmentation pattern impacts downstream immune response to the tumor

**FIGURE 6:**
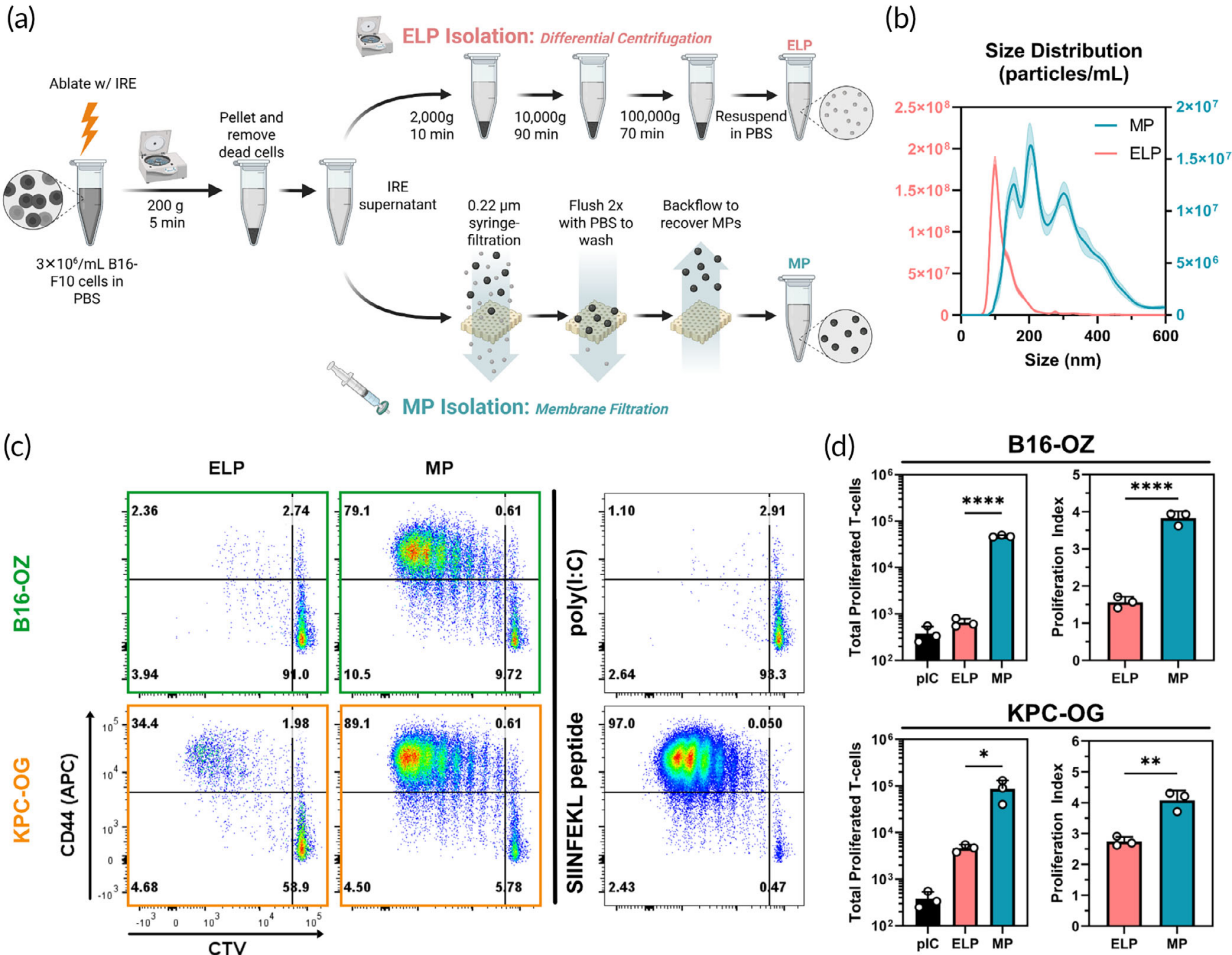
IRE‐derived microparticles (MPs) elicit increased T‐cell proliferation compared to exosome‐like particles (ELP) in vitro on a per particle basis. (a) ELP and MP isolation from IRE supernatants. ELPs were isolated via differential centrifugation and MPs were isolated via membrane filtration. (b) Size distributions of representative ELP and MP preparations as determined by NTA. Line represents mean and shaded area represents SEM. (c) Representative dot plots displaying T cells from in vitro T‐cell proliferation assay. T cells were incubated for 72 h with DCs that were pulsed with 5 mg/mL poly(I:C) and equal numbers of MPs or ELPs from IRE‐treated B16‐OZ or KPC‐OG cells for 24 h. Particles were administered at a ratio of 5.83 × 10^3^ particles per DC. As a negative control, DCs were treated only with 5 mg/mL poly(I:C). As a positive control, DCs were pulsed with 0.167 pg/mL of the OVA peptide SIINFEKL. (d) Total number of proliferated T cells and proliferation index (average number of cell divisions for the CTV^low^ cells) for the in vitro T‐cell proliferation assays in (c). Error bars represent SD, *n* = 3. Data is from one representative experiment of three independent experiments. Statistical analysis performed using unpaired *t* tests: *p < 0.05, **p < 0.01, ****p < 0.0001.

Although ELPs were the most numerous particle type generated by each ablation method, we hypothesized that the less numerous, but unique, population of MPs resulting from IRE may be supplying a superior immunogenic signal given the enhanced uptake observed with IRE supernatant. To test this hypothesis, we designed an experiment to compare how each particle type would affect downstream T‐cell proliferation while controlling for particle number. ELPs and MPs were isolated from IRE supernatants using differential centrifugation and membrane filtration, respectively (Figure 6a), and their size and concentration were quantified with NTA (Figure 6b). Then ELPs or MPs were added to BMDCs at equal particle number along with poly(I:C) for overnight incubation. Finally, naïve CTV‐labeled OT‐I T cells were added for an additional 72 h, after which the T cells were counted and assessed for proliferation (Figure S13); for each cell division the fluorescence intensity of the daughter cells decreases by a factor of roughly two. For both B16‐OZ cells and KPC‐OG cells, MPs stimulated significantly more T‐cell proliferation compared to an equal number of ELPs (Figure 6c). B16‐OZ MPs led to 73 times as many proliferated daughter T cells compared to ELPs, and KPC‐OG MPs led to 19 times as many daughter T cells as ELPs (Figure 6d). Additionally, the proliferation index, a measure of how many cell divisions the average daughter cell has undergone, was greater for MPs than ELPs: 3.8 ± 0.2 vs. 1.6 ± 0.2 for B16‐OZ, and 4.1 ± 0.3 vs. 2.7 ± 0.2 for KPC‐OG.

**FIGURE 7:**
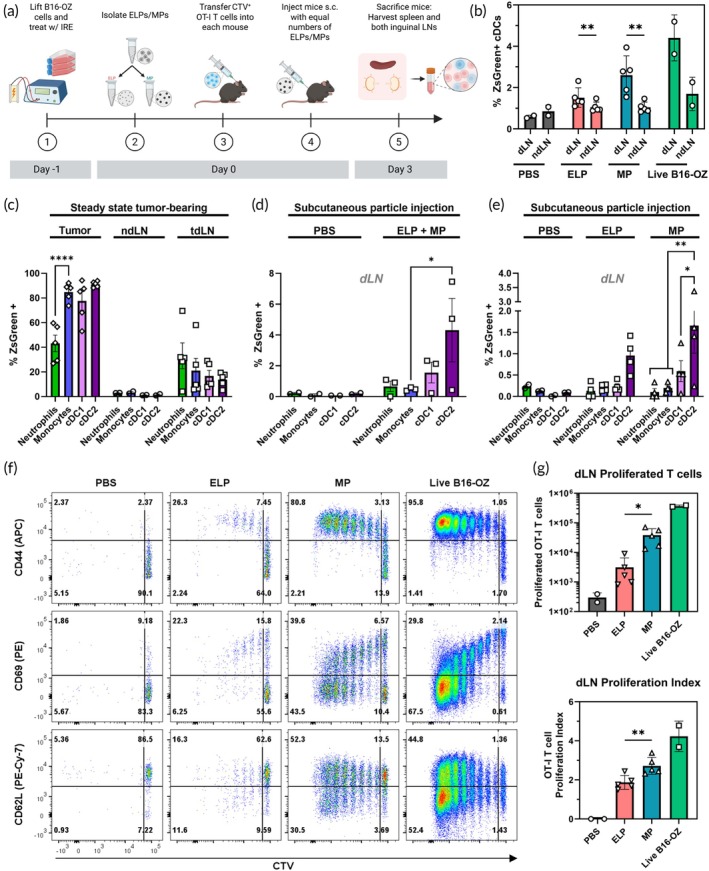
IRE‐derived microparticles (MP) elicit increased T‐cell proliferation compared to exosome‐like particles (ELP) in vivo on a per particle basis. (a) C57BL/6 mice with adopted CTV‐stained OT‐I cells were injected with equal numbers of ELPs or MPs derived from IRE‐treated B16‐OZ cells, and after 3 days the inguinal LNs were harvested and processed. OT‐I proliferation in response to the OVA‐carrying particles was assessed by observing the dilution of CTV in the OT‐I daughter cells. (b) Percent of DCs in the draining lymph node (dLN) or non‐draining lymph node (ndLN) that stained positive for ZsGreen. For negative and positive controls, mice were injected with PBS or 5 × 10^5^ live B16‐OZ cells, respectively. Bars represent mean ± SD. (c) ZsGreen uptake in various myeloid populations in the tumor, ndLN, and tumor dLN of mice bearing subcutaneous B16‐OZ tumors. (d, e) ZsGreen uptake in various myeloid populations in the dLN of naïve mice injected subcutaneously with (d) all submicron particles (MP + ELP combined) or (e) ELPs and MPs separately isolated from IRE‐treated B16‐OZ cells. (c–e) Bars represent mean ± SEM. (f) Representative dot plots characterizing activation markers of OT‐I cells harvested from the dLN as well as their degree of proliferation. (g) Total number of daughter T cells (CTV^low^) for each condition as well as their proliferation index (average number of cell divisions for the CTV^low^ cells). Bars represent mean ± SD. Statistical analysis for (b–e) was performed using two‐way ANOVA with Tukey's test for multiple comparisons, and analysis for (g) was performed using unpaired *t* tests: *p < 0.05, **p < 0.01, ****p < 0.0001. Each data point represents one mouse.

To determine if this result could be recapitulated in vivo, naïve CTV‐stained OT‐I T cells were transferred into C57BL/6 mice, which were then injected subcutaneously with equal numbers of ELPs or MPs isolated from IRE‐treated B16‐OZ cells (Figures 7a and S14). As negative and positive controls, mice were injected with PBS or live B16‐OZ cells, respectively. After 72 h the inguinal lymph nodes and spleens were harvested from the mice and analyzed with flow cytometry to determine the amount of antigen (ZsGreen‐OVA) present in various myeloid populations, as well as the degree of proliferation of the OT‐I T cells. For mice inoculated with either MPs or ELPs, there was a significantly higher percentage of ZsGreen^+^ cDCs in the draining lymph node (dLN) compared to the non‐draining lymph node (ndLN) (Figure 7b). There was also a trend toward a higher percentage of ZsGreen^+^ cDCs in the dLNs of mice inoculated with MPs compared to ELPs. To contextualize the pattern of antigen drainage from injected submicron particle debris within the broader framework of antigen drainage from an established tumor, we analyzed ZsGreen uptake in various dLN myeloid cells of B16‐OZ tumor‐bearing mice. In a steady‐state tumor model (Figure 7c), the neutrophil, monocyte, cDC1, and cDC2 populations in the tumor dLN all contained similar amounts of ZsGreen. However, in naïve mice receiving an injection of IRE‐derived submicron debris (Figure 7d), ZsGreen uptake was biased toward the cDC populations, particularly cDC2s; 4.3% ± 3.6% of cDC2s and 1.6% ± 1.2% of cDC1s were positive for ZsGreen compared to 0.7% ± 0.6% and 0.5% ± 0.1% of neutrophils and monocytes, respectively. When this experiment was repeated with ELPs and MPs administered individually, the same trends held (Figure 7e). Mice receiving ELPs displayed ZsGreen only in the cDC2 population whereas mice receiving MPs contained a ZsGreen^+^ cDC1 population as well. Additionally, within the dLNs, particle‐derived antigen was found almost exclusively in the MHCII^hi^, CD11c^int^ migratory cDC population,^40^ and little to no antigen was found in the MHCII^int^, CD11c^hi^ resident cDC population (Figure S15), indicating drainage for these particles is cell‐mediated and not passive. Thus, it appears as though ZsGreen^+^ submicron particles are preferentially trafficked to the dLN by migratory cDCs and not by other myeloid populations, which normally participate in antigen drainage of the more complex TME. It should be noted that macrophages are also expected to traffic these submicron particles, although we did not explicitly stain for them.

Regarding the effect of submicron particles on T‐cell expansion, all proliferated (CTV^low^) OT‐I T cells of the dLN showed increased expression of CD44 and PD‐1, markers of antigen experience (Figures 7f and S16). However, mice inoculated with MPs displayed 12 times greater OT‐I T‐cell expansion in the dLN compared to mice inoculated with ELPs, and these cells possessed a proliferation index of 2.7 ± 0.4 compared to 1.9 ± 0.4 for ELP‐treated mice (Figure 7g). Additionally, in comparison to ELP‐treated mice, proliferated T cells from MP‐treated mice showed decreased expression of CD69 and CD62L at higher generations (Figure 7f). This decrease is indicative of the conferral of lytic, effector functions^41^ and is consistent with an earlier initial activation, allowing for egress of the T cells from the dLN at this timepoint. Indeed, MP‐treated mice, but not ELP‐treated mice, contained a small but significant population of CD44^+^, CD69^−^ proliferated OT‐I T cells in the spleen and non‐draining inguinal lymph node, indicating dissemination of these cells out of the dLN into systemic circulation (Figure S16). Thus, for both in vitro and in vivo systems, MPs engender greater T‐cell proliferation and a more progressed immune response compared to ELPs on a per particle basis.

## 4 DISCUSSION

This study demonstrates that the biochemical composition of cell debris from IRE treatment regulates antigen processing and activation of DCs, therefore impacting the quality of the downstream CD8^+^ T‐cell immune response. These impacts are traced to both the amount and biophysical context of antigen and DAMPs released from IRE‐treated cells, including the degree of cellular fragmentation and the physical size of the resulting debris. Our findings reveal a previously unappreciated significance of this morphological aspect of IRE treatment. We show that a defining characteristic of IRE‐treated cancer cells is gross cellular fragmentation leading to the presence of two categories of particulate debris distinct from temperature‐based ablations: 2–6 μm LDPs as well as 200–1000 nm MPs. These particle subsets are both generated during the application of high magnitude pulsed electric fields, which nucleate hydrophilic pores in the cell membrane. This compromises membrane integrity and can lead to blebbing and even total cellular rupture. Several in vitro studies have shown that as a result, cytosolic contents are leaked into the extracellular space, including organelles, macrovesicles containing tumor antigen, chromatin‐containing debris, or whole nuclei.^11^, ^42^, ^43^, ^44^

To investigate if these cellular events also occurred during in vivo treatment, we analyzed tumor cell death kinetics and fragmentation patterns following IRE treatment of KPC tumors in a murine model. While cell fragmentation occurred on the order of minutes for KPC cell suspensions treated in vitro, cell death responses appeared over a 24‐h period after treatment in an in vivo setting. This longer time scale may be due to the structural context of the connected tissue or limitations in the perceived dose experienced by tissues and cells in vivo. Adjacent cells and extracellular matrix provide structural support for the cell that prevents instantaneous, gross fragmentation. One of the first observations in vivo was cellular discohesion, which was observable as early as 15 min post‐IRE. This is consistent with previous reports showing treatment of adherent cells with pulsed electric fields induced cell retraction from their spindle‐like morphology into a spherical shape.^42^, ^45^ This was tracked to calcium‐mediated loss of focal adhesions and disruption to the F‐actin cytoskeleton network.^42^, ^43^, ^45^ Similarly, we note that by 24 h post‐IRE, cellular discohesion progressed to a more severe tissue disaggregation where cells became completely detached from surrounding cells, underwent shedding of cell fragments, and released their DNA into the extracellular space. Despite its delayed onset, cellular fragmentation appears to be a previously underappreciated feature of IRE treatment in vivo, which merits further examination in preclinical models.

The first class of debris particles unique to IRE is LDPs, which we showed to contain notable immunostimulatory molecules. For example, IRE‐derived LDPs contain tumor antigen, the “eat‐me” signal HSP70,^46^ and agonists of the DC‐activating STING pathway like dsDNA.^47^ This is consistent with a known dependence of anti‐tumor CD8^+^ T‐cell priming on extracellular DNA sensing via the STING pathway in intratumoral DCs, which were activated and produced IFN‐β in response to observed tumor DNA uptake in the TME.^31^ Further studies in mouse models ranging from cancer to obesity have shown that the cDC1 subpopulation is predominantly responsible for taking up extracellular DNA from MPs or apoptotic bodies and producing inflammatory cytokines and chemokines (e.g., CXCL9, type‐I interferons, IL‐12) in response to activation of the STING pathway.^48^, ^49^ Thus, it is likely that a similar role for cDC1s might be implicated in the immune response to IRE tumor ablation, where numerous DNA‐containing LDPs are released, and indeed we did see a highly biased uptake of tumor DNA in the cDC1 population in vitro. However, further studies are needed to show a causal relationship between IRE‐released DNA and STING activation in these DCs. Additionally, we showed that a significant number of the LDPs created by IRE contain plasma membrane. Recent reports have shown that cholesterol accessibility can activate DCs, and intracellular cholesterol obtained by uptake of cellular debris is vital for the generation of lipid nanodomains that DCs use to amplify T‐cell priming.^50^ Finally, our results confirmed that antigen‐bearing LDPs were internalized by DCs in accordance with their abundance, with IRE leading to greater uptake than Heat or Cryo.

The second class of particulate debris unique to IRE is MPs. Tumor‐derived MPs generated by UV radiation,^51^, ^52^, ^53^ membrane extrusion,^54^ and sonication^55^, ^56^ have been shown to protect against tumor challenge and inhibit metastasis via DC activation when administered as a vaccine. Because of this evidence for successful MP‐based immunomodulation, we used subcellular fractionation to enrich MPs and ELPs from IRE‐treated tumor cells in order to compare their immunogenicity. This revealed that MPs were superior to ELPs in the induction of CD8^+^ T‐cell proliferation in vitro and in vivo. Regarding the role of cDCs, we observed that migratory cDCs were responsible for transporting the particles to the dLN, as minimal LN‐resident DCs contained antigen. Literature has pointed to cDC1s as the dominant subset for uptake^57^, ^58^, ^59^ and transport^35^, ^48^, ^60^ of tumor debris larger than 100 nm to the dLN. Although our in vivo model demonstrated that draining lymph node cDC2s appeared to take up more submicron antigen than cDC1s (Figure 7e), the cDC1s showed increased uptake of MPs compared to ELPs, suggesting a possible size dependence for uptake. This is consistent with the observation that cDC1s were the most profound acceptor for the much larger LDPs in vitro. Additionally, it should be noted that our uninflamed in vivo model of ELP and MP injection contrasts with the immunosuppressive TME, which has been shown to suppress cDC2 migration to the dLN via Tregs.^61^ Indeed, for our model of steady state KPC‐OG tumor drainage, we observe antigen positivity in cDC1s is just as high as cDC2s.

The ability to produce cellular fragmentation may be a key delineator of the immunogenicity of focal ablation modalities. In contrast to IRE, other common ablation methods did not cause fragmentation that resulted in significant numbers of >200 nm particles. For Heat, this is likely because of protein denaturation and coagulation caused by the increased temperature. Even if a more rapid and intense heating methodology was applied, such as microwave ablation used clinically, aggregation would likely prevent particle dissemination as coagulation necrosis is predominantly observed in the ablation zone for numerous animal models.^62^ Although minor fragmentation was observed under Cryo treatment, and particle generation could plausibly be increased further with more freeze–thaw cycles or a faster cooling rate, clinical application of IRE, but not Cryo or Heat, leaves important physiological structures (blood/lymphatic vessels, bile ducts, extracellular matrix, etc.) intact. This facilitates faster DC and T‐cell migration to and from the site of ablation.^11^, ^12^, ^21^, ^63^, ^64^ Thus, for improved DC antigen uptake, not only must particulate antigen be generated, but it must also be accessible to infiltrating immune cells. Conversely, histotripsy, which ablates cells with ultrasound‐mediated acoustic cavitation, may be a focal therapy that shares IRE's fragmentation pattern and also preserves critical structures.^65^ In vitro treatment of cancer cells with histotripsy produces a debris field with subcellular fragments very similar to IRE, as well as the release of antigen and immunostimulatory molecules like HMGB1, HSP70, and calreticulin at levels comparable to other non‐thermal modalities.^66^, ^67^, ^68^, ^69^, ^70^ This leads to successful activation of dLN resident cDCs in murine models.^71^ Compared to ablation methods that do not share a fragmented debris morphology, like heat and radiation, histotripsy‐treated tumors contained greater numbers of infiltrated DCs and CD8^+^ T cells, and inoculation of mice with ablation supernatants from ex vivo treated cancer cells imparted partial protection from tumor challenge, which was not seen for heat or radiation supernatants.^67^, ^72^ This suggests that a pattern of cellular fragmentation is a broader phenomenon not exclusive to IRE, and other ablation methods that create such a cell debris morphology by different means, while still maintaining a tissue architecture that allows for cellular migration, would likely benefit from the same immunogenic effects.

Likewise, it can also be said that optimization of IRE parameters (e.g., field strength, pulse width, pulse number, waveform, etc.) to maximize the degree of cell fragmentation can further boost its immunological benefits to improve patient outcomes clinically. As we observed, increasing the IRE field strength directly enhanced the release of extracellular DNA and submicron particles from treated cancer cells, both of which have the potential to increase DC function. Additionally, higher field strengths have been proven to significantly increase the size of the ablation zone, allowing clinicians to treat larger tumors.^73^ However, increasing field strength too much (via high voltage pulses) leads to unwanted heat generation, which can coagulate and destroy sensitive nearby structures like nerves and bile ducts.^74^ Thus, this study provides rationale for clinicians to select IRE parameter sets that increase cellular fragmentation for improved immunogenicity.

## 5 CONCLUSIONS

Here we demonstrate that the debris morphology of IRE‐treated cancer cells impacts DC uptake of antigen and DAMPs and subsequent CD8^+^ T‐cell proliferation, an aspect that has received little attention previously. Compared to the temperature‐mediated ablative modalities tested here, IRE produced a significant increase in LDPs, which were shown to contain antigen and dsDNA, leading to increased uptake of these molecules by DCs. Additionally, IRE generated more submicron MPs, which resulted in greater T‐cell proliferation and activation compared to ELPs. These findings unlock new opportunities for the improvement of future ablation‐based in situ cancer vaccine designs through parameters that lead to increased cellular fragmentation.

## AUTHOR CONTRIBUTIONS

**Joseph R. Vallin**: Conceptualization, Investigation, Formal analysis, Methodology, Validation, Writing – original draft, Writing – review and editing. **Brandon Burbach**: Conceptualization, Investigation, Methodology, Writing – review and editing, Resources. **Qi Shao**: Methodology, Resources, Writing – review and editing. **Fang Zhou**: Investigation, Resources, Writing – review and editing. **Jacob S. Ankeny**: Conceptualization, Writing – review and editing. **Alessio Giubellino**: Investigation, Methodology, Writing – review and editing. **Yoji Shimizu**: Resources, Writing – review and editing. **Samira M. Azarin**: Project administration, Supervision, Conceptualization, Funding acquisition, Writing – review and editing.

## FUNDING STATEMENT

This work was supported by the University of Minnesota.

## CONFLICT OF INTEREST STATEMENT

The authors declare no conflicts of interest.

## Supporting information


**Data S1:** Supporting information.

## Data Availability

The data that support the findings of this study are available from the corresponding author upon reasonable request.
