# Supplementary material for: Cellular fragmentation underlies the immunogenicity of irreversible electroporation‐mediated tumor cell killing
Source: Bioeng Transl Med. 2025 Dec 22;11(3):e70102. doi: 10.1002/btm2.70102 (PMC13247414; doi:10.1002/btm2.70102)
Supplement: Supplementary file 1 — Data S1: Supporting information. [file BTM2-11-e70102-s001.pdf]

Supplementary Information

Cellular Fragmentation Underlies the Immunogenicity of Irreversible Electroporation-Mediated Tumor Cell Killing

Joseph R. Vallin, Brandon J. Burbach, Qi Shao, Fang Zhou, Jacob S. Ankeny, Alessio Giubellino, Yoji Shimizu, Samira M. Azarin

Contents:

1. Supplementary Methods
2. Supplementary Table 1
3. Supplementary Figures S1-S16

Supplementary Methods

Focal ablation of adherent cells

For treatment of adherent cells with IRE, cells were grown in tissue culture treated 6-well plates in complete media until they reached 80-90% confluency. Then the cells were washed twice with PBS and 2 mL of serum free media was added to each well. A 2D-culture electroporation applicator (Petri Pulser, BTX) was inserted into each well and either left idle for 100 seconds (sham control) or used to administer a dose of 1250 V/cm with 50 µs pulse width, 99 pulses, and 1 Hz. After treatment, cells were incubated at 37 ºC and imaged with EVOS FL Auto microscope at ~30 min or 24 h post treatment.

ImageJ quantification of cell debris size and number

Brightfield micrograph images were taken of each ablation lysate using an EVOS FL Auto microscope (ThermoFisher Scientific). ImageJ software was used to count and determine the size of each particle in the ablation lysates. Briefly, images were converted to 8-bit and the background was subtracted. Then a bandpass filter was applied, and the images were manually thresholded into a binary image. The Watershed and Fill Holes functions were used to clean up the image along with manual image correction to fix obvious errors in the thresholding. Finally, the Analyze Particles function was applied to count the particles and determine their area and Feret's diameter.

Immunostaining procedure

Samples were pelleted onto SuperFrost™ Plus microscope slides (Fisherbrand) with a Shandon Cytospin 4 cytocentrifuge (ThermoFisher Scientific) for 3 min at 1000 RPM. Immediately after pelleting the sample, a circle was drawn around each sample with a hydrophobic marker and 100 µL of 4% paraformaldehyde (PFA) (Sigma) fixative was placed on each sample for 15 min at room temperature. Next, the slides were washed 3 times with PBS and then blocked

for 60 min at room temperature with 50 µL of blocking buffer containing 0.3% (v/v) Triton-X 100 (Sigma) and 5% (v/v) normal goat serum (Sigma) in PBS. After removing the blocking buffer, samples were incubated overnight with 50 µL of primary antibody staining solution composed of 1% BSA (w/v) (Sigma), 0.3% Triton-X 100, and primary antibody (see **Table S1**) in PBS at 4˚C in a humidified chamber. Samples were washed in PBS 3 times and then incubated for 60 min in a dark humified chamber with 50 µL of secondary antibody staining solution composed of 1% BSA (w/v), 0.3% Triton-X 100, and 1:1000 goat anti-rabbit IgG AlexaFluor 647 conjugate in PBS. Next, the samples were washed with PBS 3 times and then incubated for 10 minutes in the dark with 50 µL of DAPI diluted at 1:1000 in PBS. Slides were washed with PBS and then mounted with 1 drop of SlowFade Glass Soft-set Antifade Mountant (Invitrogen). Coverslips were sealed onto slides using CoverGrip sealant (Biotium). Samples were imaged using an EVOS FL Auto microscope or an A1Rsi HD confocal microscope (Nikon) equipped with 405 nm, 488 nm, and 640 nm lasers to view DAPI, ZsGreen, and AlexaFluor647 respectively.

Isolation of Splenic DCs

The spleens of mice with B16-Flt3L flank tumors were harvested, placed in harvest buffer (RPMI-1640 with 5% FBS, 10 mM HEPES and 4mM L-glutamine), and minced with scissors. The volume of harvest buffer was brought to 1.8 mL and 0.2 mL of 10x collagenase D (Sigma) was added (400 U/ml final concentration), followed by a 30 min incubation at 37 ˚C. Digested spleens were mashed though a 70 µm filter, and the resulting single cell suspension was washed with harvest buffer and resuspended in 1.5 mL of MojoSort buffer (BioLegend) in a sterile FACS tube. 7.5 µL each of 0.5 mg/ml biotin anti-Thy1.2, biotin anti-CD19, biotin anti-Ter119, and biotin anti-NK1.1 antibodies (Cytek Tonbo Biosciences) were added to 150 µL of rat serum (STEMCELL Technologies), which was then mixed with the splenocytes and incubated for 10 min at room

temperature. Then, 100 μL of MojoSort streptavidin magnetic beads (BioLegend) were added and incubated for a further 5 min. Splenocytes and beads were diluted with an additional 1 mL of MojoSort buffer, placed in a magnet (STEMCELL Technologies) for 3 min, and decanted into TCPM. Isolated DCs were spun down at 1500 RPM for 5 min, resuspended in TCPM, and stored on ice until they were ready to be used.

Isolation of natural EVs for TEM imaging

B16-F10 cells at ~50% confluency were washed with PBS, and serum free media was added for 20 hours after which the cells were fully confluent. The conditioned media was collected and EVs were isolated via differential centrifugation (300 g for 10 min and 2,000 g for 20 min) to remove dead cells and large debris. The EVs were then pelleted at 100,000 g for 70 min and resuspended in ~100 μL of PBS.

OT-I T cell isolation

LNs and spleen were mashed through a 70 μm cell strainer to form a single cell suspension. The cells were then washed in TCPM and resuspended in 1 mL of RoboSep Buffer (STEMCELL Technologies) in a FACS tube where they were mixed with 50 μL of rat serum (STEMCELL Technologies), 2.5 μL of biotin anti-CD44 antibody, and 50 μL of a CD8 negative separation antibody cocktail (see **Table S1**). After 10 min of incubation, 100 μL of MojoSort streptavidin nanobeads were mixed in and the suspension was incubated for 5 more min. Then, the suspension was diluted to 2.5 mL with RoboSep buffer, placed in a magnet for 3 min, and then decanted into 10 mL of TCPM.

Supplementary Table 1: Antibody List

Conjugate	Target (mouse)	Dilution	Vendor
**Immunostaining**			
**Primary**	TRP2	1:200	Invitrogen
**Primary**	HSP70 (hu)	1:200	ProteinTech
**DNA Uptake**			
BUV395	Siglec H	1:100	BioLegend
BV421	CD11b	1:100	BioLegend
BV510	MHCII	1:1000	BioLegend
BV650	CD11c	1:100	BioLegend
BV711	CX3CR1	1:100	BioLegend
BV785	XCR1	1:100	BioLegend
PerCp-Cy5.5	F4/80	1:100	BioLegend
FITC	CD3	1:100	BioLegend
PE	CD86	1:100	BioLegend
AF700	CD45	1:100	BioLegend
Fixable Viability Dye eFluor™ 780	--	1:1000	Invitrogen
**ZsGreen Uptake**			
BUV395	CLEC9A	1:100	BD
BV421	CD11b	1:100	BioLegend
BV510	MHCII	1:1000	BioLegend
Super Bright 600	CD103	1:100	Invitrogen
BV650	CD11c	1:100	BioLegend
BV785	XCR1	1:100	BioLegend
PE	CD86	1:100	BioLegend
PerCP-Cy5.5	Sirp-α	1:100	BioLegend
APC	Siglec H	1:100	BioLegend
AF700	CD45	1:100	BioLegend
Fixable Viability Dye eFluor™ 780	--	1:1000	Invitrogen
**PKH26 Uptake**			
BV421	CD86	1:100	BioLegend
BV510	MHCII	1:1000	BioLegend
Super Bright 600	CD103	1:100	Invitrogen
BV650	CD11c	1:100	BioLegend
BV785	XCR1	1:100	BioLegend
FITC	F4/80	1:100	BioLegend
PerCP-Cy5.5	Sirp-α	1:100	BioLegend
APC	Siglec H	1:100	BioLegend
AF700	CD45	1:100	BioLegend
Fixable Viability Dye eFluor™ 780	--	1:1000	Invitrogen

**Splenic DC magnetic separation**			
Biotin	CD19	--	Tonbo Biosciences
Biotin	Ter119	--	Tonbo Biosciences
Biotin	NK1.1	--	Tonbo Biosciences
Biotin	Thy1.2	--	Tonbo Biosciences
**T cell magnetic separation**			
Biotin	IA/IE	--	BioLegend
Biotin	CD45R/B220	--	Tonbo Biosciences
Biotin	CD4	--	Tonbo Biosciences
Biotin	CD19	--	Tonbo Biosciences
Biotin	NK1.1	--	Tonbo Biosciences
Biotin	F4/80	--	Tonbo Biosciences
Biotin	Ly6G	--	Tonbo Biosciences
Biotin	CD16/32	--	Tonbo Biosciences
Biotin	CD44	--	Tonbo Biosciences
**In vitro T cell activation assay**			
BV605	MHCII	1:1000	BioLegend
PE-Cy7	CD44	1:100	BioLegend
BV711	CD8a	1:100	BioLegend
APC	CD69	1:100	BioLegend
PerCP-Cy5.5	Thy1.1	1:200	BioLegend
Fixable Viability Dye eFluor™ 780	--	1:1000	Invitrogen
**In vivo IRE submicron particle injection experiment**			
BUV395	Thy1.1	1:200	BD
BUV496	B220	1:100	BD
BUV737	CD8a	1:100	BD
BV510	Sirp1α	1:100	BioLegend
BV605	PD-1	1:100	BioLegend
BV650	XCR1	1:100	BioLegend
BV711	CX3CR1	1:100	BioLegend
BV785	Thy1.2	1:200	BioLegend
PerCP-Cy5.5	Ly6G	1:100	BioLegend
PE	CD69	1:100	BioLegend
PE-Dazzle594	CD11c	1:100	BioLegend
APC	CD44	1:100	BioLegend
AF700	MHCII	1:100	BioLegend
Fixable Viability Dye eFluor™ 780	--	1:1000	Invitrogen

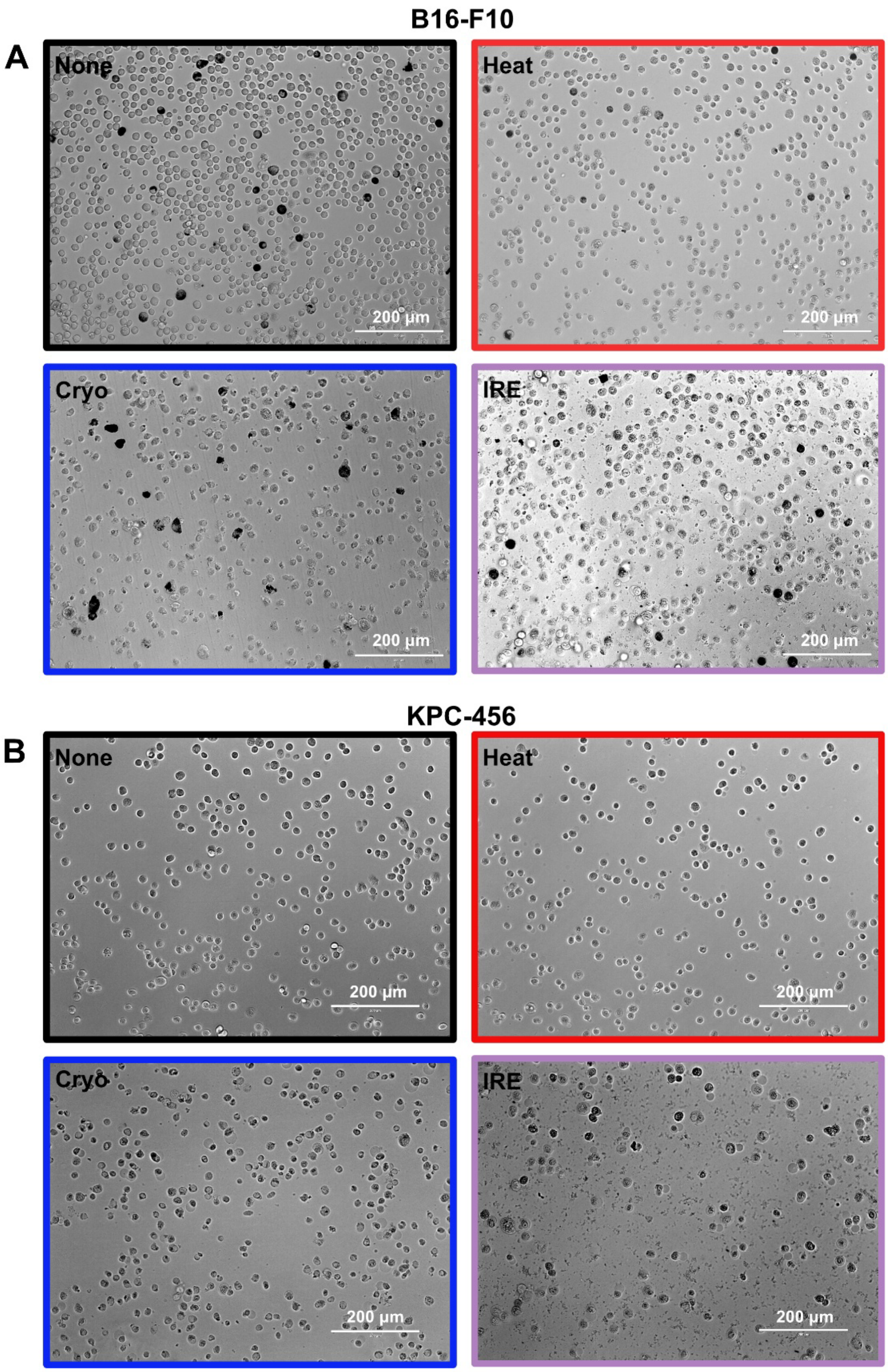

Figure S1: **Light microscopy of focally ablated cancer cells**. Representative image (20x magnification) of each ablation method applied to (A) B16-F10 cells and (B) KPC-456 cells. Four independent images for each condition were used to quantify the size distribution of the cellular debris using ImageJ. Scale bar: 200 µm.

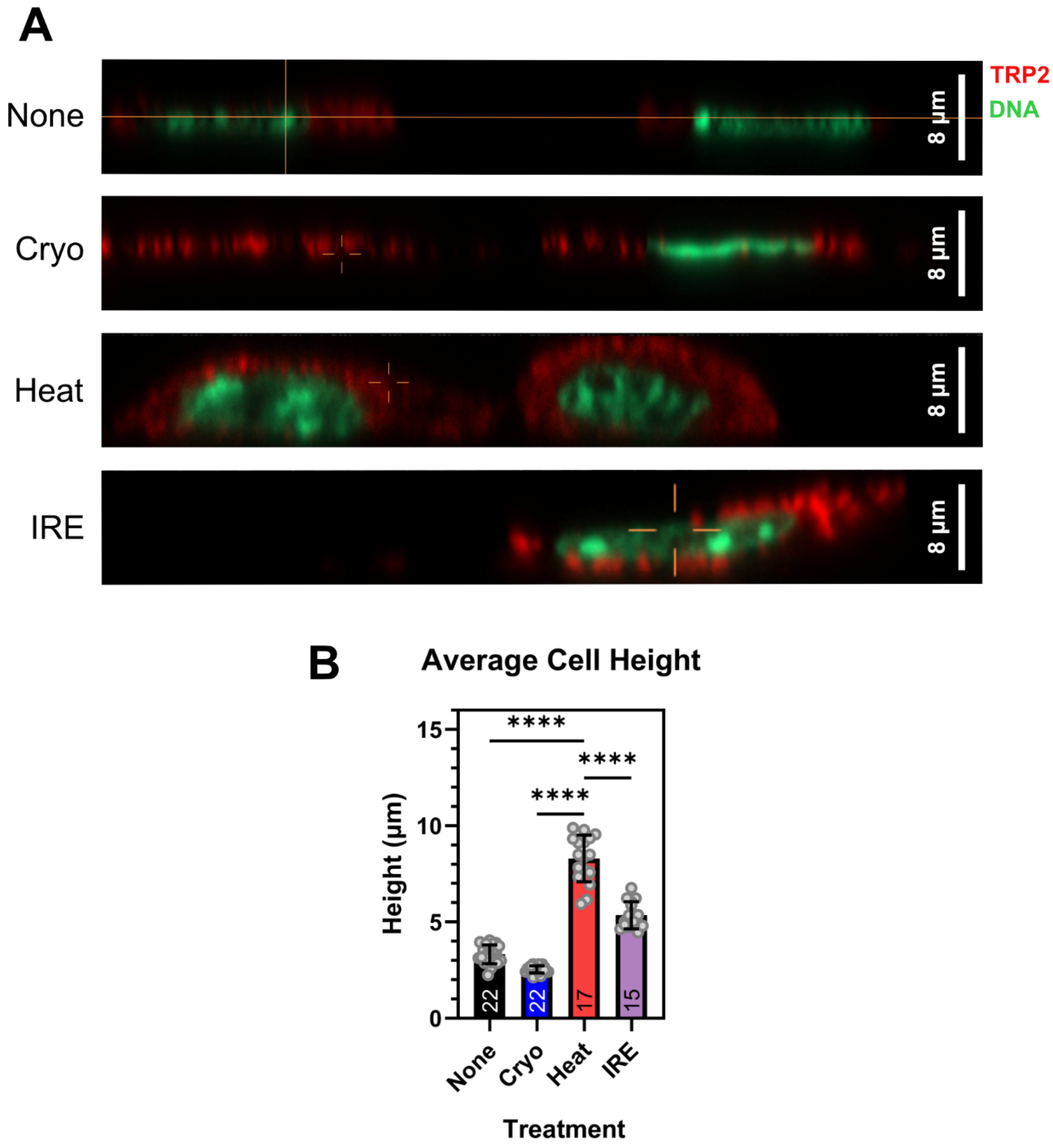

Figure S2: **Heat-treated cells maintain a greater height after centrifugation**. (A) Z-stack projections of ablated B16-F10 cells and an untreated control (None) that were pelleted with a cytocentrifuge at 2,000 RPM for 3 min onto glass slides. Cells were immunostained to visualize TRP2 (AF647) and DNA (DAPI). Scale bar = 8 µm. (B) Average cell height was calculated by taking 15-22 measurements (n listed in bars) across the length of the cells in each image using ImageJ. Error bars represent SD. Statistics performed by taking a Brown-Forsythe and Welch ANOVA test with Dunnett T3 test for multiple comparisons. **** $p < 0.0001$

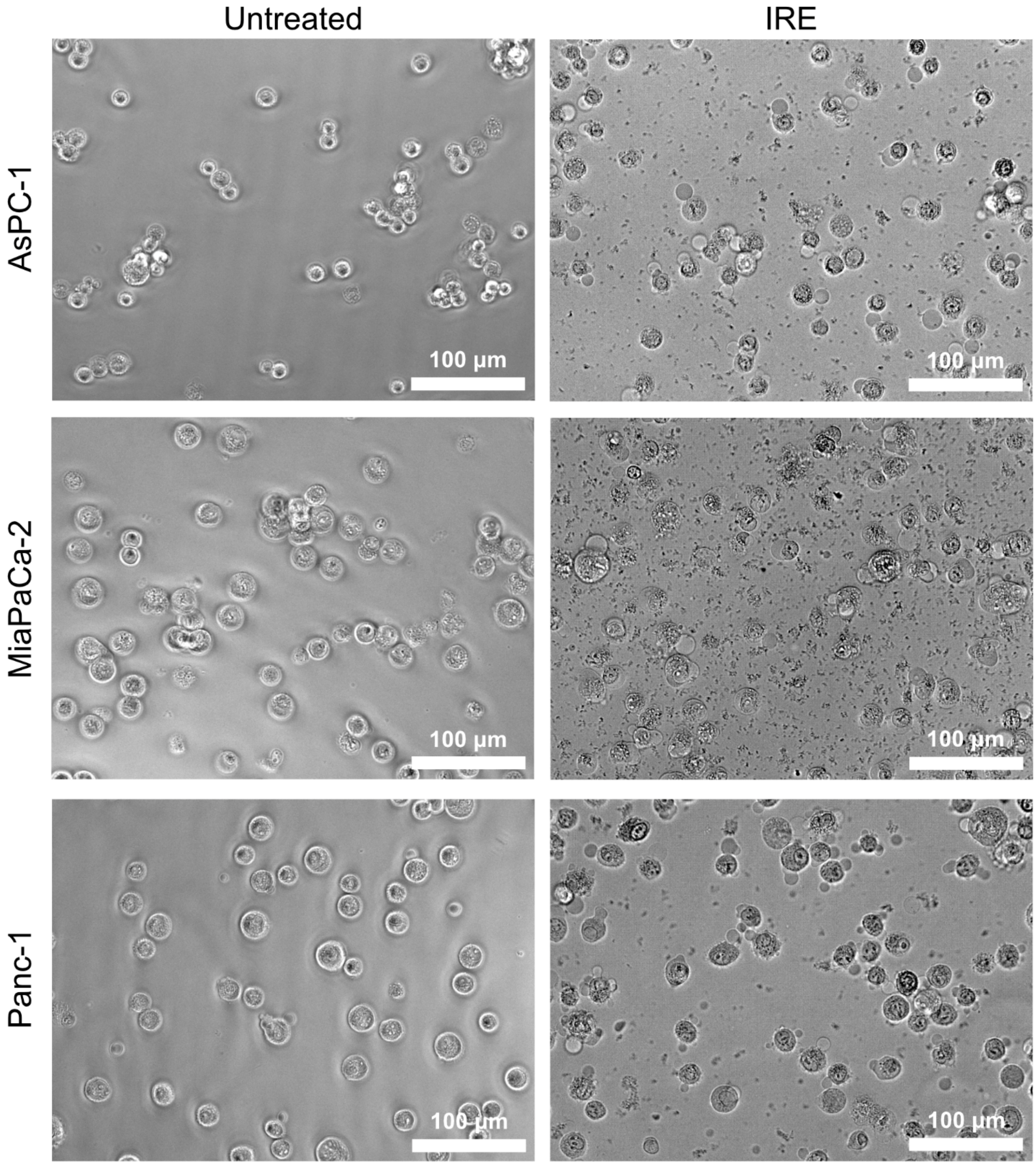

Figure S3: **Brightfield micrographs of IRE-treated human PDA cell lines show presence of cellular fragmentation and LDP generation**. AsPC-1, MiaPaCa-2, and Panc-1 cells were suspended in PBS to 1×10^6 cells/mL and then treated with IRE (1250 V/cm, 50 µs pulse, 99 pulses, and 1 Hz). Scale bar: 100 µm.

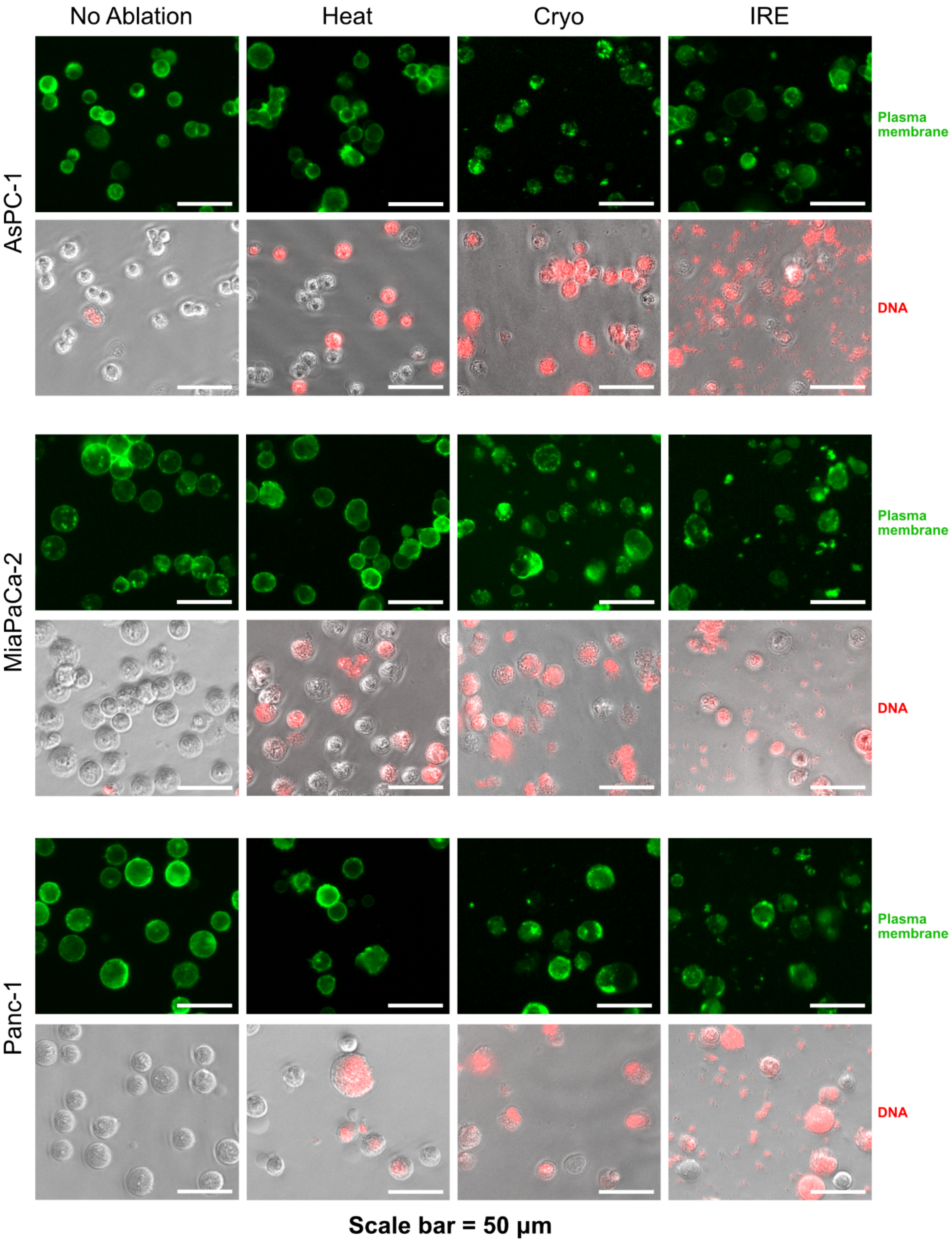

Figure S4: **Biochemical characterization of focally ablated human PDA cell lines reveals plasma membrane and DNA on LDPs**. Cells in PBS suspension of 1×10^6 cells/mL were treated with Heat, Cryo, or IRE. Plasma membrane was stained with wheat germ agglutinin (WGA) and DNA was stained with ethidium homodimer-1 (EthD-1). DNA immunostaining was superimposed over phase micrographs of the samples. Scale bar: 50 µm.

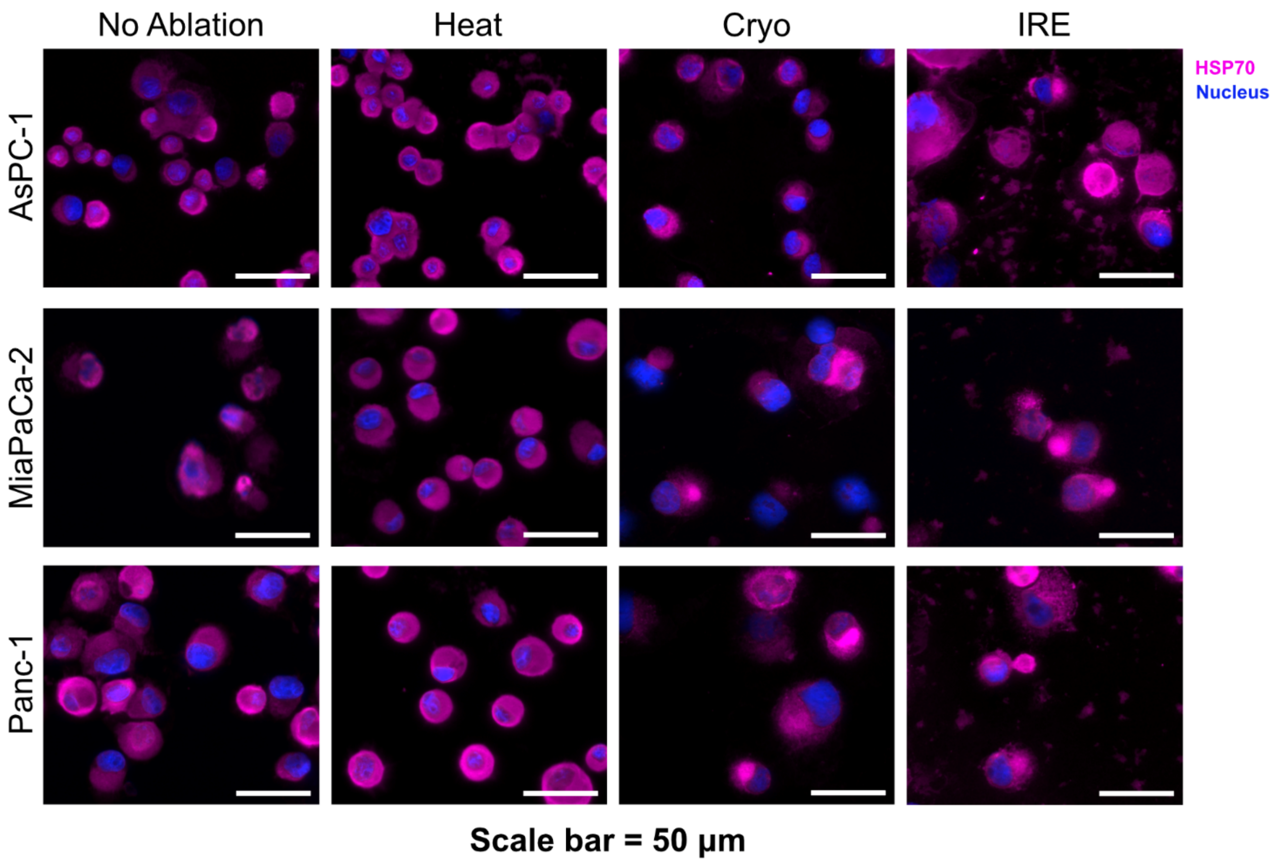

Figure S5: **HSP70 is found on LDPs of IRE-treated PDA cell lines**. Cells in PBS suspension of 1×10^6 cells/mL were treated with Heat, Cryo, or IRE. Nuclei were counterstained with DAPI, and HSP70 was stained with primary rabbit anti-HSP70, followed by AF647 labeled secondary goat anti-rabbit IgG. Scale bar: 50 µm.

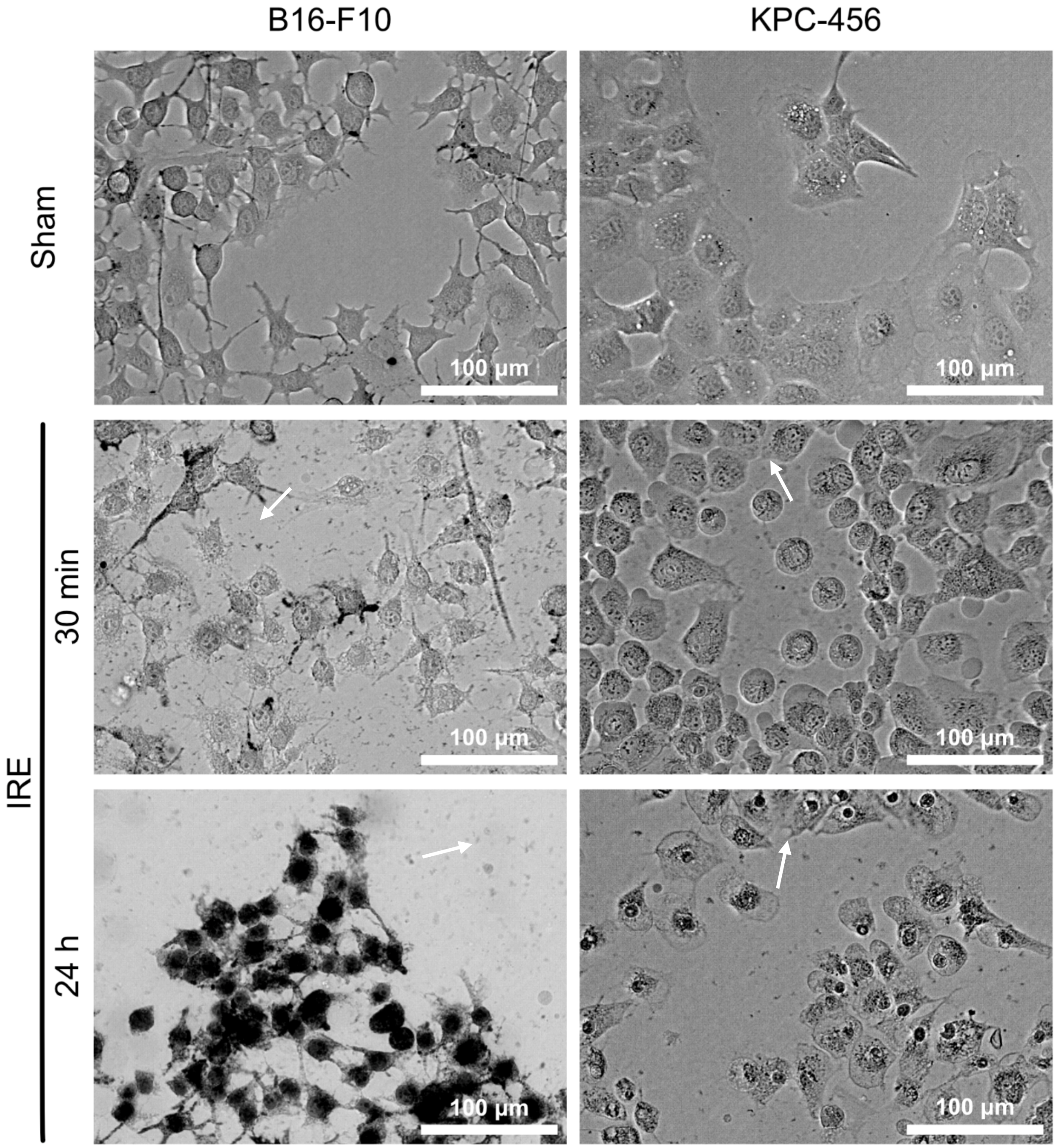

Figure S6: LDPs are generated by adherent B16-F10 and KPC-456 cells after IRE treatment. B16-F10 and KPC-456 cells were grown to 80-90% confluency in 2D culture and then treated with IRE (1250 V/cm, 50 µs pulse width, 99 pulses, and 1 Hz) or a sham treatment negative control. Representative light microscopy images are shown of cells approximately 30 min or 24 h after treatment. LDPs are observed in the extracellular space for treated samples (some examples are indicated with arrows). Scale bar: 100 µm.

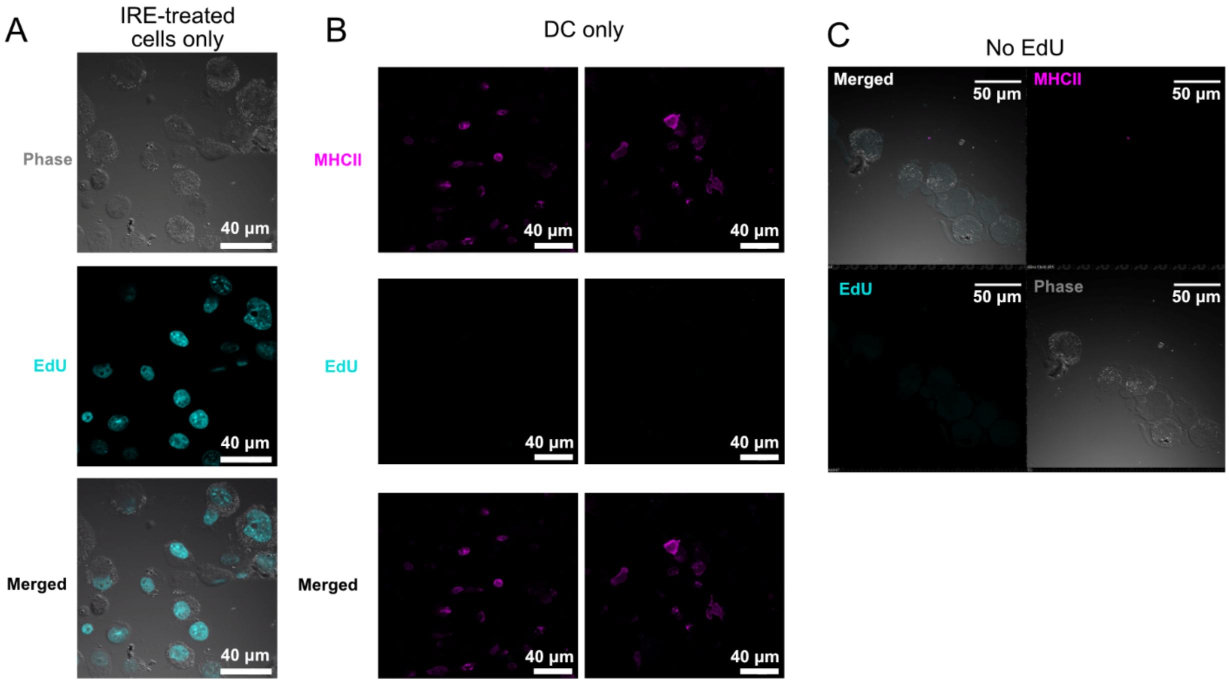

Figure S7: Immunostaining controls for DC uptake of cancer cell-derived DNA. (A) EdU immunostaining of IRE-treated B16-F10 cells alone. Scale bar: 40 µm. (B) EdU immunostaining of BMDC only control. Scale bar: 40 µm. (C) EdU immunostaining of negative EdU control: B16-F10 cells not treated with EdU (but still stained with azide-AF647). Scale bar: 50 µm. All images were obtained via confocal microcopy.

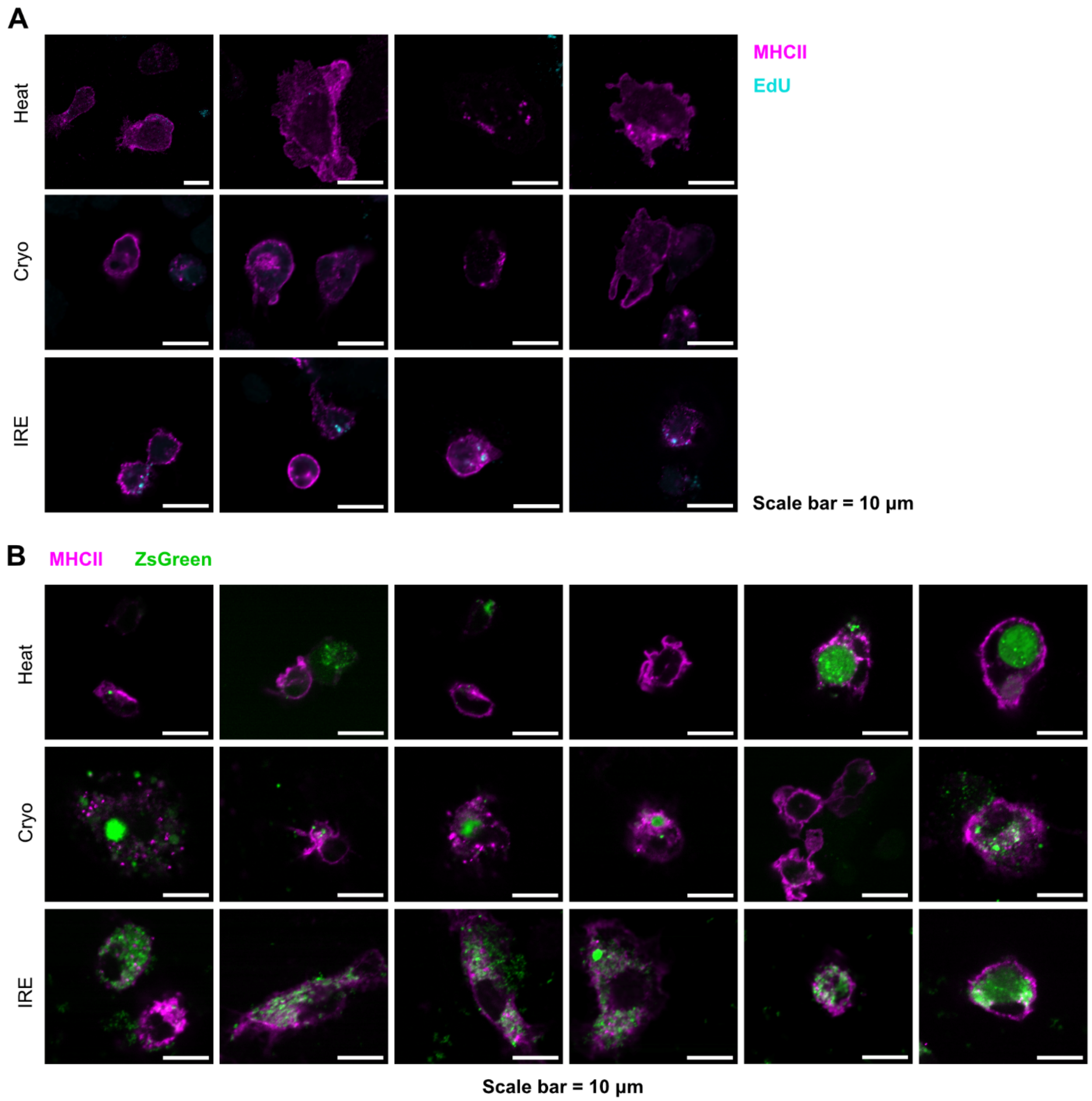

Figure S8: DC uptake of tumor derived DNA and antigen reveals increased internalization for IRE samples. Additional confocal images of BMDC uptake of (A) cancer derived EdU^+ DNA from focally ablated B16-F10 cells (n=4) and (B) ZsGreen from focally ablated KPC-ZsGreen cells (n=6). Scale bar = 10 µm.

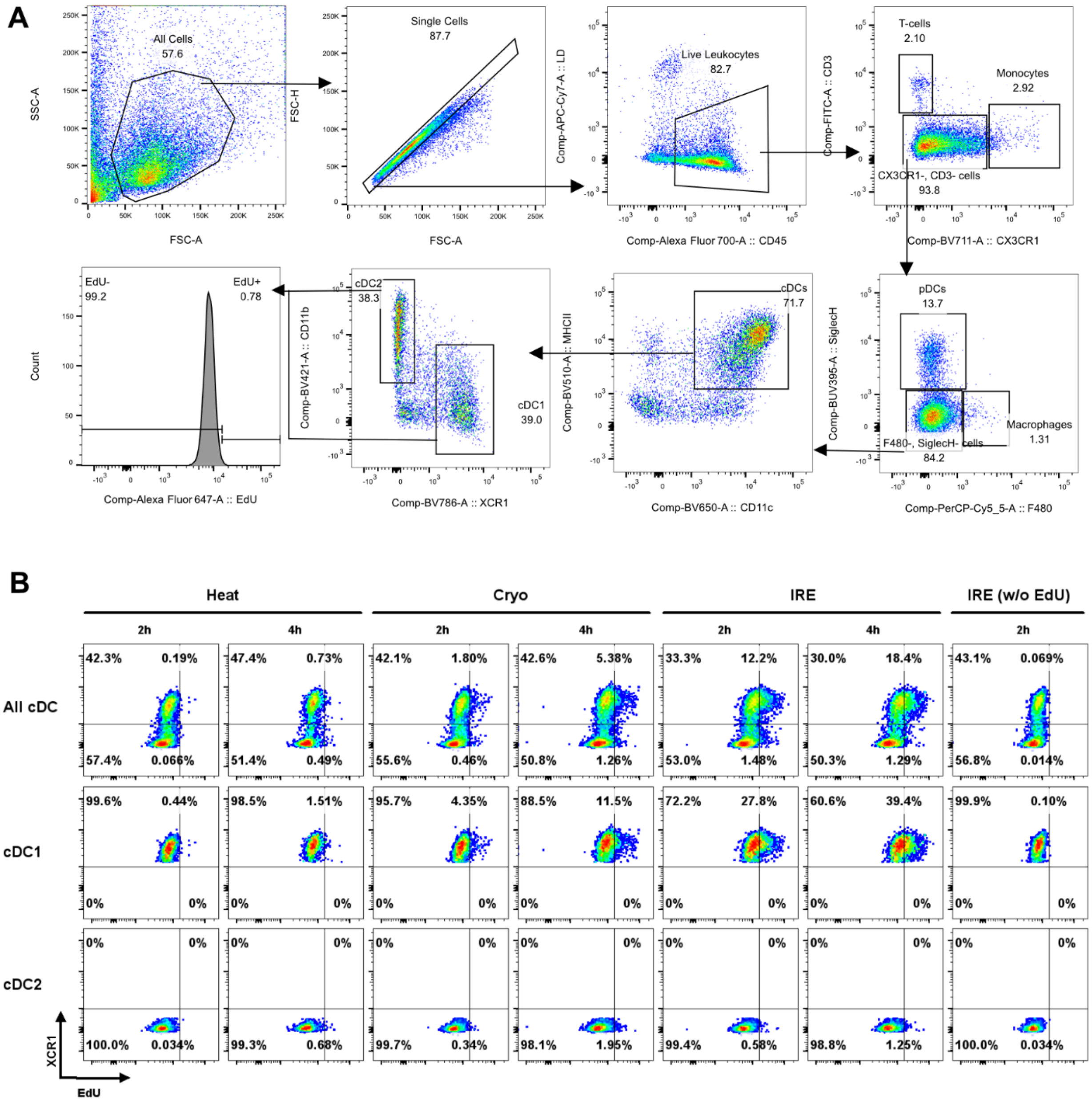

Figure S9: Measurement of splenic cDC uptake of cancer cell-derived DNA via flow cytometry. (A) Gating scheme used to identify cDCs. cDCs were defined as CD45^{+}, F4/80^{-}, Siglec H^{-}, CD11c^{+}, MHCII^{+} cells. The cDC1 subpopulation was identified as XCR1^{+}, Sirp-α^{-} whereas the cDC2 subpopulation was identified as XCR1^{-}, Sirp-α^{+}. (B) Representative dot plots of splenic DC uptake of EdU (AF647)-labeled B16-F10-derived DNA from ablation supernatants. The gate for EdU positivity was set using DCs incubated with IRE-treated B16-F10 cells that were not treated with EdU.

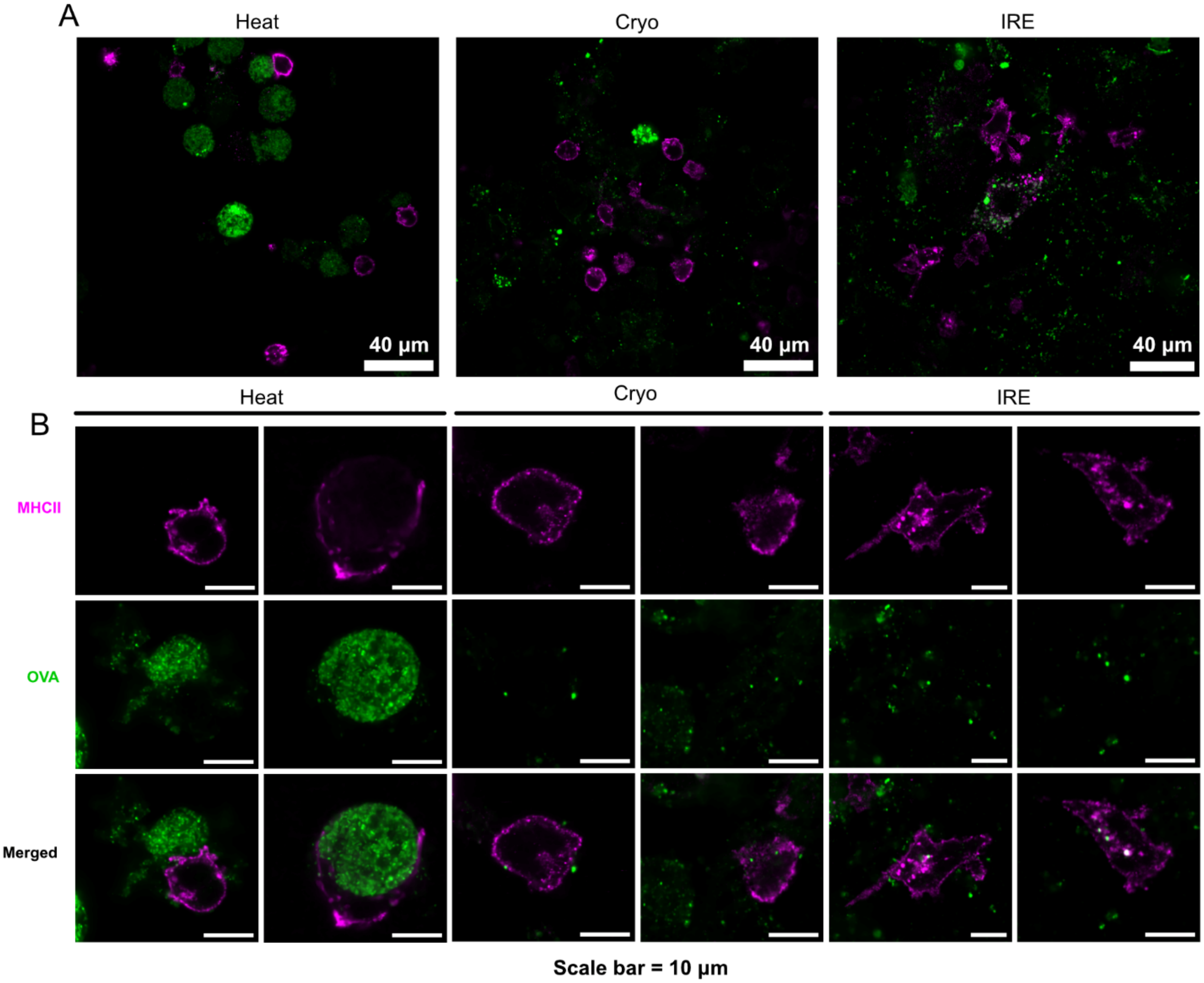

Figure S10: DC internalization of OVA antigen from ablated B16-OZ cells is greatest for IRE treated cells. (A) Immunostaining of BMDCs incubated with focally ablated ZsGreen-OVA-expressing B16-OZ cells for 3 hours. Scale bar: 40 µm. (B) Two representative high magnification images of BMDC uptake of OVA from focally ablated B16-OZ cells after a 3-hour co-incubation. Scale bar: 10 µm. All images were obtained via confocal microcopy.

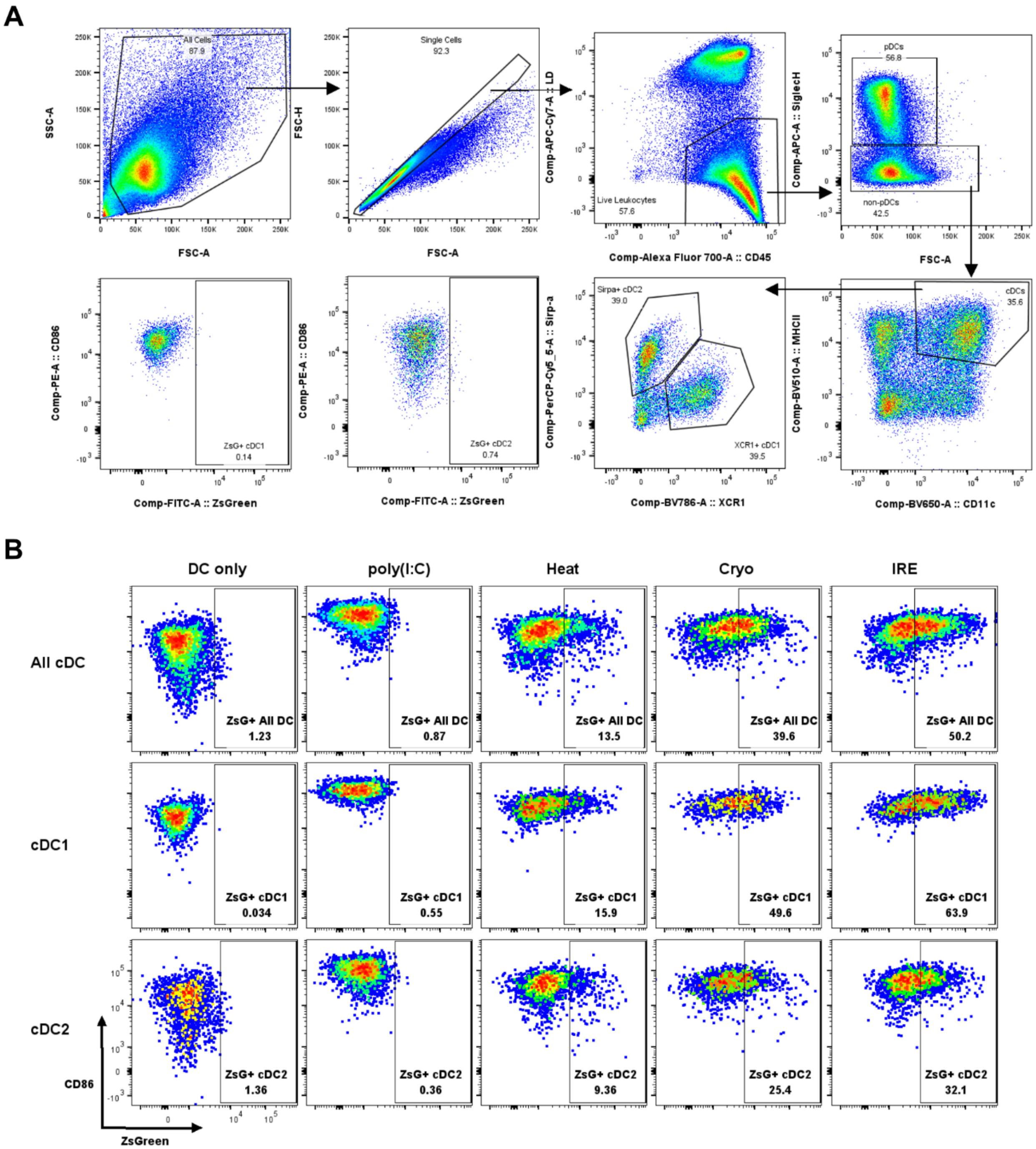

Figure S11: **Measurement of cDC uptake of proxy antigen (ZsGreen) from focally ablated KPC-ZsG cells via flow cytometry**. (A) Gating scheme used to identify cDCs. Anti-Siglec H was used to gate out pDCs. (B) Representative dot plots of DC uptake of ZsGreen from ablated KPC-ZsG cells. The gate for ZsG positivity in cDCs was set using the control condition where DCs were incubated without ablated cells (DC only).

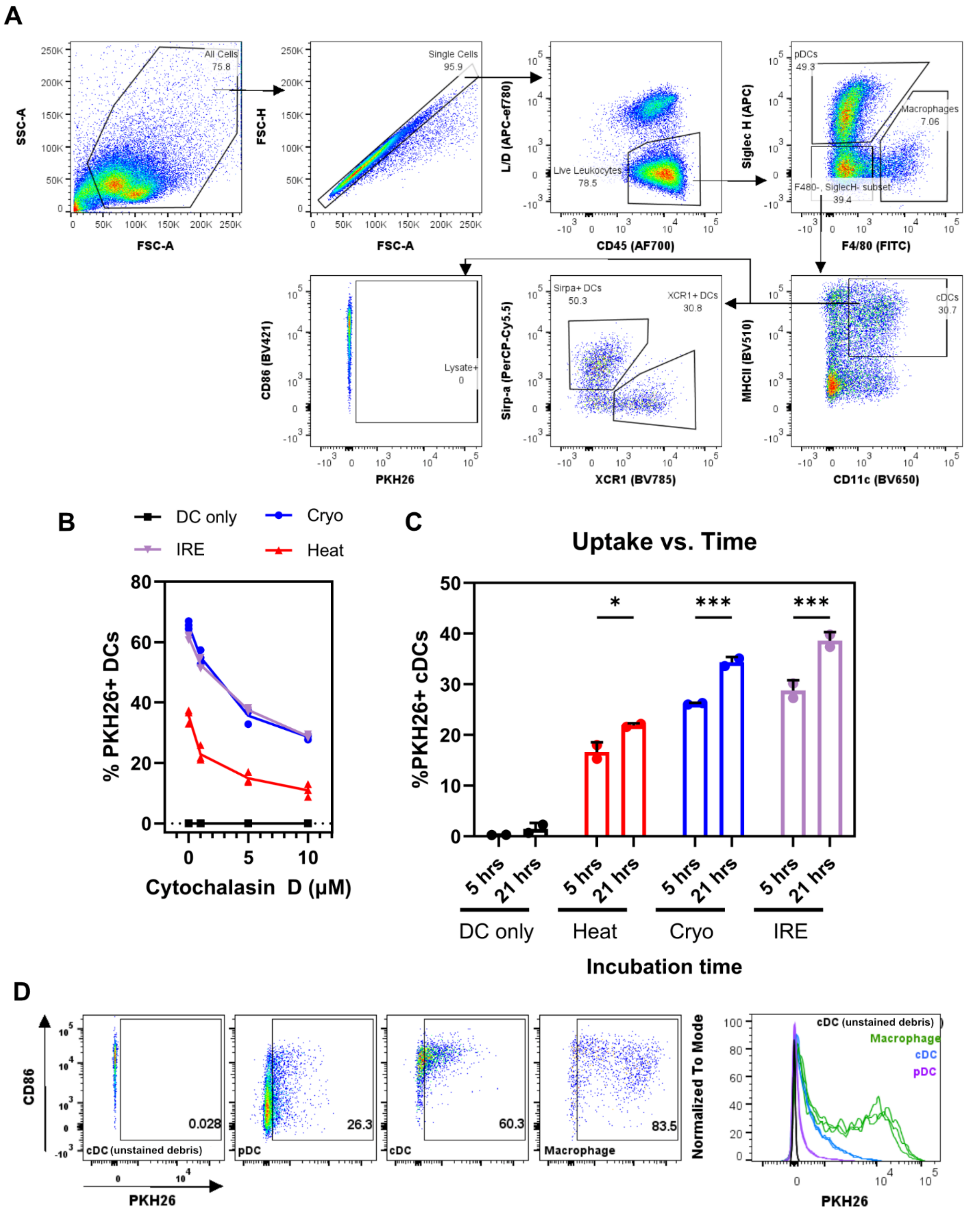

Figure S12: **Validation of the flow cytometry-based BMDC uptake assay using PKH26-stained B16-F10 cells.** (A) Gating scheme showing the identification of cDCs. Siglec H and F4/80

expression were used to gate out pDCs and macrophages, respectively. cDCs were identified as CD11c$^+$, MHCII$^+$ cells. The positive gate for PKH26 positivity in cDCs was drawn on cDCs incubated with IRE-treated, unstained B16-F10 cells. (B) Uptake of cell debris from ablated B16-F10 cells by cDCs after 3 hours of co-incubation, where cDCs were treated with increasing amounts of the phagocytosis inhibitor cytochalasin D. Line passes through the mean values; n = 3. Data from one of two representative independent experiments. (C) B16-F10 debris uptake in cDCs after 5 and 21 hours of co-incubation with ablated cells. Error bars denote SD; n = 2. Data from one of two representative independent experiments. Statistical analysis was performed using Tukey's test for multiple comparisons: * $p < 0.05$, *** $p < 0.001$. (D) Representative dot plots showing uptake of IRE B16-F10 debris by cDCs (CD11c$^+$, MHCII$^+$), pDCs (Siglec H$^+$), and macrophages (F4/80$^+$) after 3 hours of co-incubation. Histogram shows uptake of three technical replicates from a single experiment representative of 3 independent experiments.

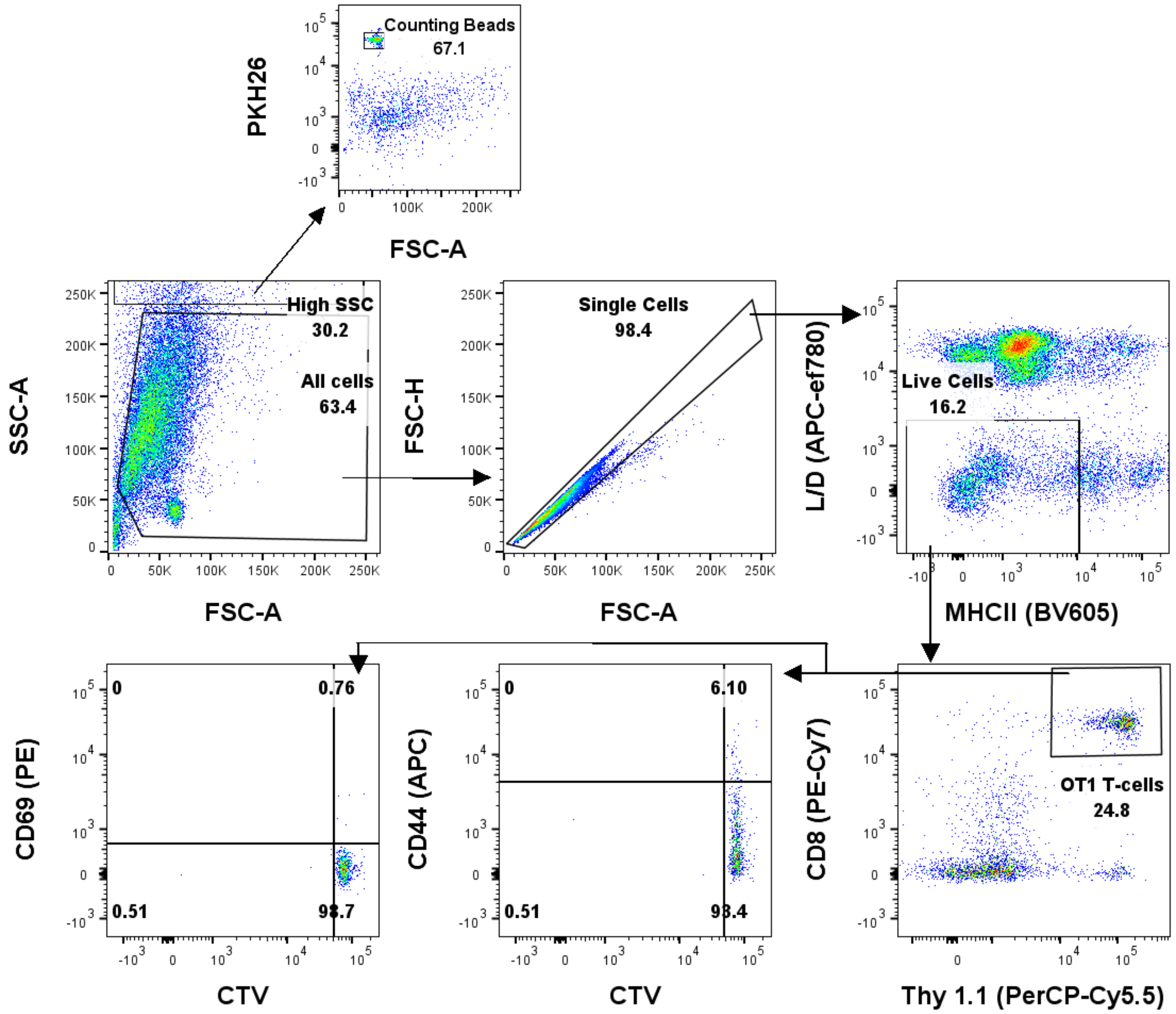

Figure S13: Gating scheme for *in vitro* T cell proliferation assay. Donor OT-I T cells come from mice expressing the Thy1.1 congenic marker, which is used along with CD8 to distinguish them from BMDCs. The sample shown in this gating example was a negative control of OT-I T cells and BMDCs incubated without the addition of ablated B16-OZ cells.

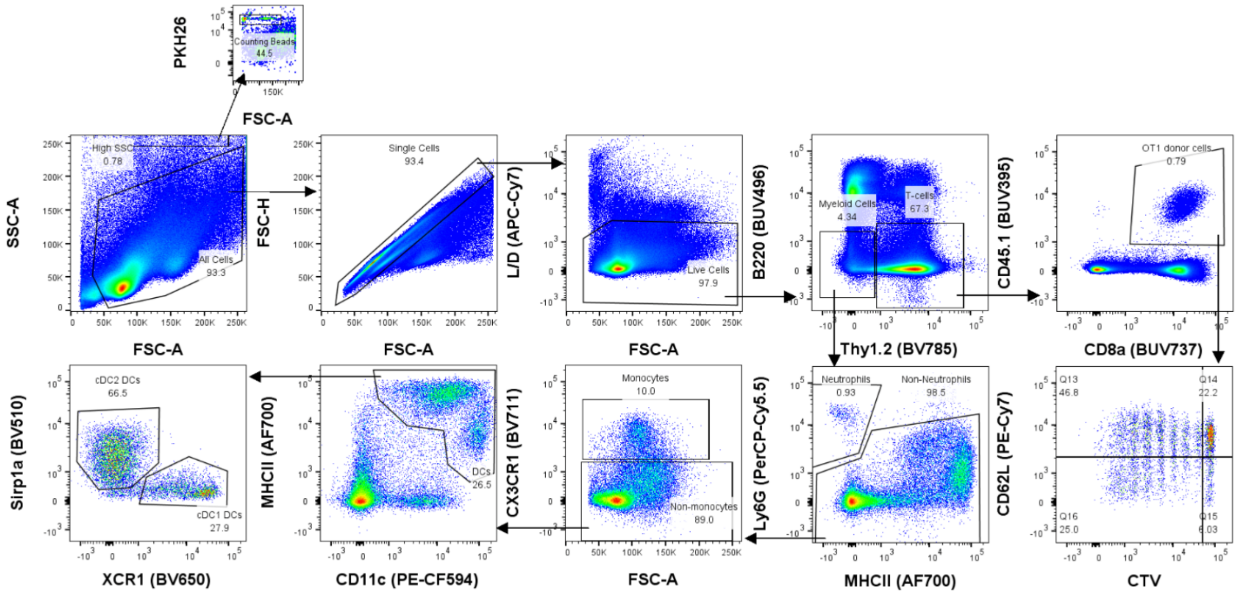

Figure S14: Gating scheme for cells harvested from mice inguinal LNs and spleen 3 days post-inoculation with B16-OZ derived MPs and ELPs. Gating scheme for identifying cDCs and OT-I cells. Briefly, B220 was used to exclude B cells, Ly6G was used to define neutrophils, and CX3CR1 was used to define monocytes. DCs were defined as MHCII^{+} and CD11C^{+}, and OT-I cells were distinguished by CD8 and CD45.1 expression, the latter of which is a congenic marker of the donor OT-I cells that was not expressed in the recipient mice.

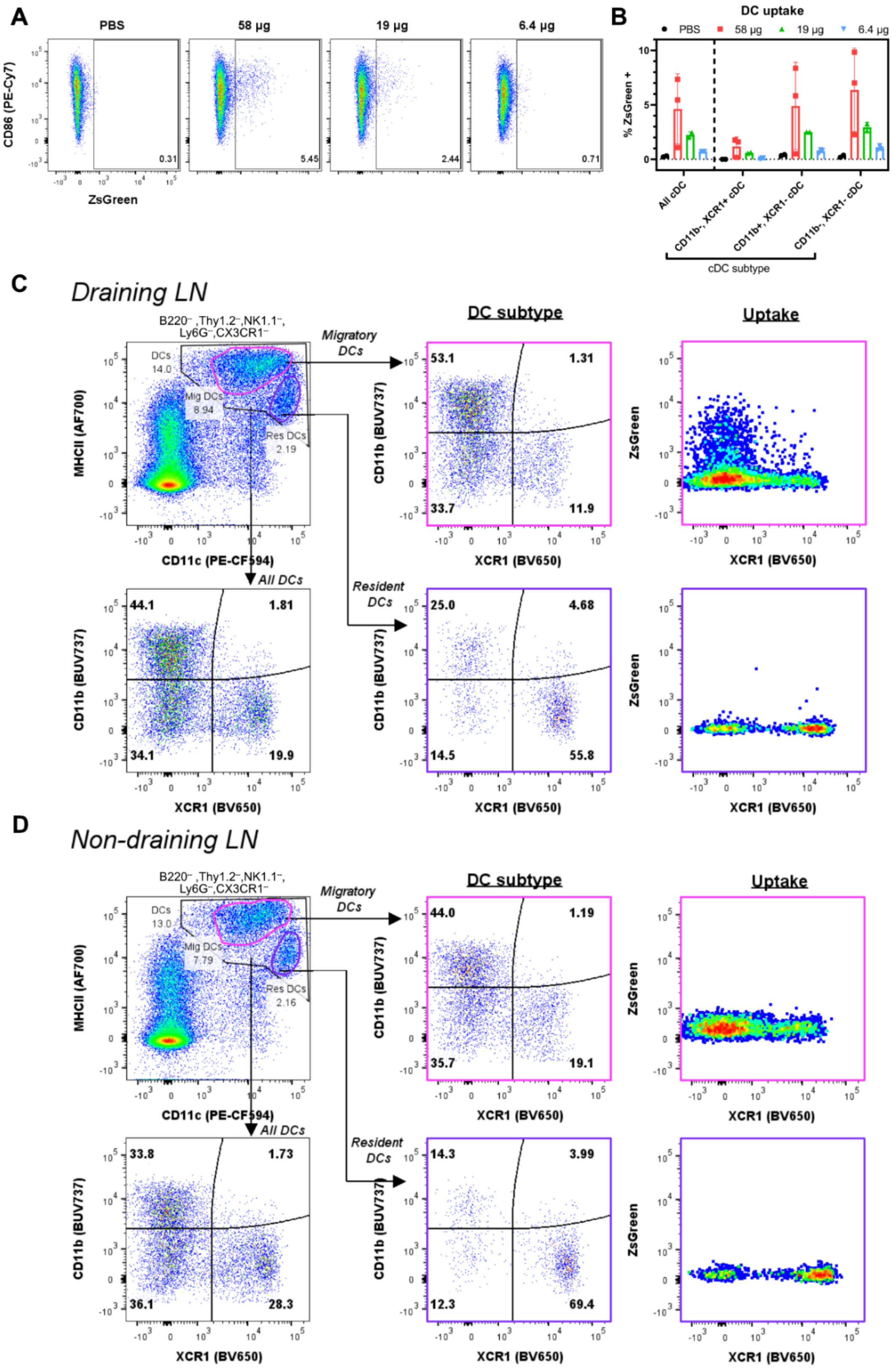

Figure S15: **IRE-derived submicron particle trafficking to the draining lymph node is dominated by migratory cDCs**. Submicron particles (MPs + ELPs) collected from the debris of

IRE-treated B16-OZ cells were injected subcutaneously into the right flanks of C57BL/6 mice at varying doses. After 48 hours, the draining and non-draining inguinal LNs were harvested and processed separately for flow cytometry. (A) Representative dot plots assessing uptake of different doses of IRE-derived submicron particles in cDCs (Siglec H-, MHCII+, CD11c+) of the dLN via ZsGreen positivity. (B) Uptake of IRE-derived submicron particles in cDCs, as well as their various subsets, in the dLN. (C) Representative characterization of the subtypes and uptake of DCs in the draining and non-draining LN of 1 of 3 mice treated with the highest titer (58 μg) of IRE submicron particles.

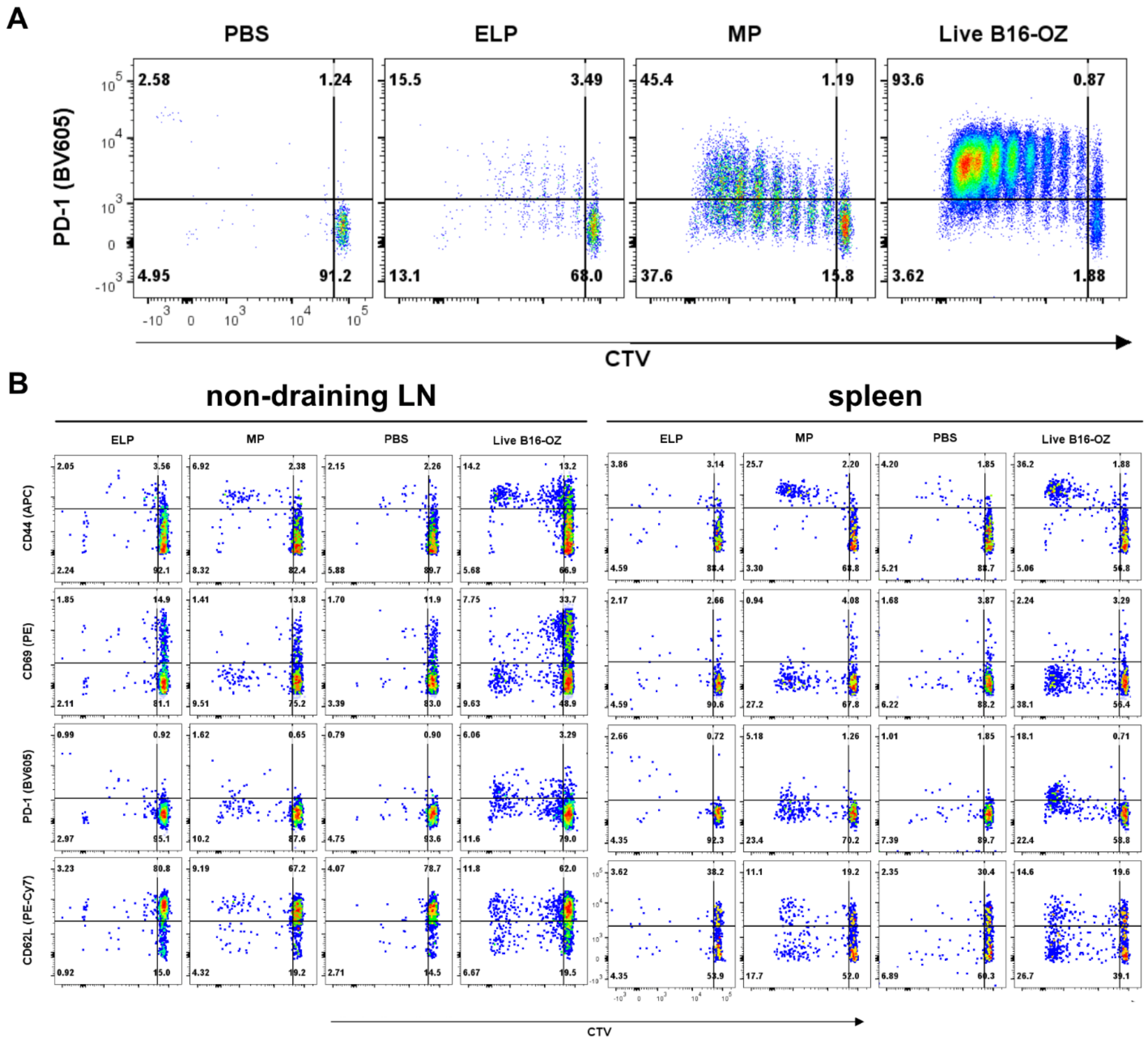

Figure S16: Additional analysis of *in vivo* OT-I T cell proliferation. (A) Representative dot plots showing PD-1 expression in OT-I T cells harvested from the dLN 3 days post injection. (B) Representative dot plots characterizing activation markers of OT-I cells harvested from the ndLN (contralateral inguinal LN) and spleen as well as their degree of proliferation.
